# Biliverdin Reductase-A integrates insulin signaling with mitochondrial metabolism through phosphorylation of GSK3β

**DOI:** 10.1016/j.redox.2024.103221

**Published:** 2024-06-01

**Authors:** Chiara Lanzillotta, Antonella Tramutola, Simona Lanzillotta, Viviana Greco, Sara Pagnotta, Caterina Sanchini, Silvia Di Angelantonio, Elena Forte, Serena Rinaldo, Alessio Paone, Francesca Cutruzzolà, Flavia Agata Cimini, Ilaria Barchetta, Maria Gisella Cavallo, Andrea Urbani, D. Allan Butterfield, Fabio Di Domenico, Bindu D. Paul, Marzia Perluigi, Joao M.N. Duarte, Eugenio Barone

**Affiliations:** a Department of Biochemical Sciences “A. Rossi-Fanelli”, Sapienza University of Rome, Italy; b Department of Basic Biotechnology, Perioperative and Intensive Clinics, Faculty of Medicine and Surgery, Catholic University of the Sacred Heart, L.go F.Vito 1, 00168, Rome, Italy; c Fondazione Policlinico Universitario A. Gemelli IRCCS, L.go A.Gemelli 8, 00168, Rome, Italy; d Center for Life Nano- & Neuro-Science, Istituto Italiano di Tecnologia, 00161, Rome, Italy; e Department of Physiology and Pharmacology, Sapienza University of Rome, Italy; f Department of Experimental Medicine, Sapienza University of Rome, Italy; g Sanders-Brown Center on Aging, Department of Chemistry, University of Kentucky, Lexington, KY, USA; h The Solomon H. Snyder Department of Neuroscience, Johns Hopkins University School of Medicine, Baltimore, MD, USA; i Department of Pharmacology and Molecular Sciences, Johns Hopkins University School of Medicine, Baltimore, MD, USA; j Department of Psychiatry and Behavioral Sciences, Johns Hopkins University School of Medicine, Baltimore, MD, USA; k Lieber Institute for Brain Development, Baltimore, MD, USA; l Department of Experimental Medical Science, Faculty of Medicine, Lund University, Sweden; m Wallenberg Centre for Molecular Medicine, Lund University, Lund, Sweden

**Keywords:** Biliverdin reductase-A, Brain insulin resistance, GSK3β, Mitochondrial metabolism, Mitochondrial unfolded protein response, Oxidative stress

## Abstract

Brain insulin resistance links the failure of energy metabolism with cognitive decline in both type 2 Diabetes Mellitus (T2D) and Alzheimer's disease (AD), although the molecular changes preceding overt brain insulin resistance remain unexplored. Abnormal biliverdin reductase-A (BVR-A) levels were observed in both T2D and AD and were associated with insulin resistance. Here, we demonstrate that reduced BVR-A levels alter insulin signaling and mitochondrial bioenergetics in the brain. Loss of BVR-A leads to IRS1 hyper-activation but dysregulates Akt-GSK3β complex in response to insulin, hindering the accumulation of pGSK3β^S9^ into the mitochondria. This event impairs oxidative phosphorylation and fosters the activation of the mitochondrial Unfolded Protein Response (UPRmt). Remarkably, we unveil that BVR-A is required to shuttle pGSK3β^S9^ into the mitochondria. Our data sheds light on the intricate interplay between insulin signaling and mitochondrial metabolism in the brain unraveling potential targets for mitigating the development of brain insulin resistance and neurodegeneration.

## Highlights

• Insulin signaling alterations impairs mitochondrial metabolism in T2D brain.
• BVR-A loss alters mitochondrial bioenergetics in the brain in response to insulin.
• UPRmt is activated to face insulin signaling-mitochondria axis alterations.
• BVR-A transports GSK3β^S9^ into the mitochondria to regulate energy metabolism.
• BVR-A loss compromise the insulin response, resembling an insulin resistance state.

## Graphical abstract

**Figure:**
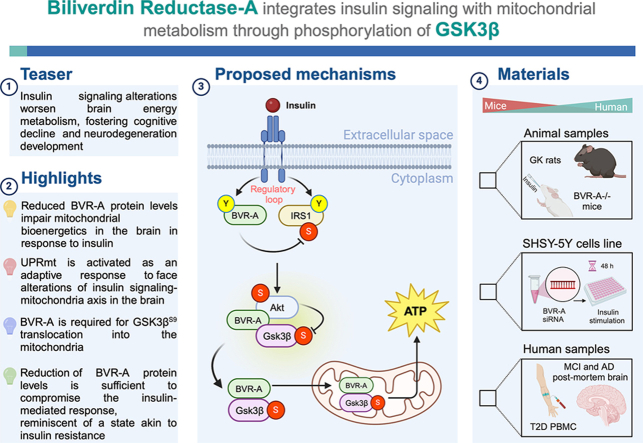

## Abbreviations

BVR-A: Biliverdin reductase-A
GSK3β: glycogen synthase kinase 3β
UPRmt: mitochondrial Unfolded Protein Response
T2D: Type 2 diabetes mellitus
AD: Alzheimer's disease
MCI: mild cognitive impairment
IRS1: Insulin receptor substrate-1
HOMA-IR: Homeostatic Model Assessment of Insulin Resistance
IPL: inferior parietal lobule
Atf5: Activating transcription factor 5
Grp75: 75-kDA glucose-regulated protein
Hsp60: heat shock protein 60
Atf4: Activating transcription factor 4
CHOP: C/EBP homologous protein
Sirt3: Sirtuin 3
Sod2: Superoxide Dismutase 2
HNE4: hydroxy-2-nonenal adducts
3-NT: 3-nitrotyrosine
OS: Oxidative stress
Gpx: glutathione peroxidase
Tfam: mitochondrial transcription factor A
Pgc1α: Peroxisome proliferator-activated receptor-gamma coactivator 1-alpha

## 1 Introduction

Type 2 diabetes mellitus (T2D) is a metabolic disorder that is closely linked to cognitive decline and poses an elevated risk of age-related cognitive issues [1]. Individuals with metabolic disorders, i.e., metabolic syndrome, obesity and T2D, face a heightened susceptibility to age-related cognitive decline, mild cognitive impairment (MCI), vascular dementia, and Alzheimer's disease (AD) [2,3]. It has been established that a broad range of cognitive domains are affected by T2D [4], which suggests a widespread impact on the central nervous system. An important link between T2D and the various forms of dementia involves alterations in the insulin signaling, particularly in the brain. Insulin resistance predicts cognitive decline, especially in individuals with prediabetes [5]. Treatments for diabetes that focus on improving insulin sensitivity might therefore have the potential to postpone or even prevent cognitive decline in patients with T2D [5]. Furthermore, intranasal insulin improves memory and cognitive functions in healthy humans, patients with AD [[6], [7], [8]] and mouse models of the disease [9]. In addition, the administration of insulin into the hippocampus improved learning ability in wild-type mice [10,11]. Remarkably, diminished insulin sensitivity at the level of the central nervous system, termed brain insulin resistance, constitutes a joint pathological feature of metabolic and cognitive dysfunction [12]. Brain insulin resistance may even develop independently from peripheral alterations [13] and negatively impact on cognitive functions by impairing the metabolic fueling of neurons [14]. Indeed, glucose metabolism and mitochondrial functions are essential ATP sources crucial for neuronal homeostasis [15]. Conversely mitochondrial dysfunction and bioenergetics deregulation contributes to defects of synaptic plasticity mechanisms which are linked to brain insulin resistance and diabetic complications [[16], [17], [18]]. From a molecular standpoint, biliverdin reductase-A (BVR-A) protein - primarily recognized for its role in the degradation pathway of heme [19] - has been identified as a key regulator of the insulin signaling pathway [20,21]. BVR-A arguably has a more diverse and expansive spectrum of functions than any other protein. This breadth of functions is imparted by its numerous consensus regulatory motifs and its ability to fold into a protein–protein interactive structure [19], notwithstanding, BVR-A is a protein with pleiotropic functions [19,22]. Through its serine/threonine/tyrosine (S/T/Y) kinase activity BVR-A regulates the insulin receptor substrate-1 (IRS1), avoiding excessive IRS1 activation in response to insulin [20,21]. Intriguingly, downstream of IRS1, BVR-A may function as a scaffold protein, and in particular, BVR-A markedly increases the pyruvate dehydrogenase lipoamide kinase isozyme 1 (PDK1)-mediated activation of the serine/threonine protein kinase B (PKB/Akt), and the Akt-mediated inhibition of the glycogen synthase kinase 3β (GSK3β) [[23], [24], [25]]. Additionally, we have shown that loss of BVR-A causes increased oxidative stress [[26], [27], [28]] and mTOR hyper-activation, which impairs autophagy leading to accumulation of oxidatively-damaged proteins [26]. Knock-out mice for BVR-A exhibited substantial deficits in learning and memory on neurocognitive tests [29], whereas increasing BVR-A protein levels in murine models for neurodegenerative disorders led to improved cognitive and learning functions [9,30], thus positioning BVR-A at a prominent intersection of synaptic plasticity mechanisms [29,31]. Previous researches have shown that reduced levels of BVR-A are strongly linked to insulin resistance in both AD brains [9,24,32] and peripheral blood mononuclear cells (PBMC) [33,34] isolated from either obese or T2D subjects [33,34], thus representing a shared mechanism. Our group also demonstrated that BVR-A alterations in T2D individuals strictly correlates with poor glycometabolic control and a pro-inflammatory state [34,35]. These lines of evidence uphold the role of BVR-A as a master regulator of insulin signaling pathway and suggest BVR-A loss as an event contributing to the onset of cellular metabolic dysfunctions. However, the molecular link among BVR-A alterations, insulin signaling and cell energy metabolism in the brain is still obscure. In the present study we prove for the first time that loss of BVR-A in the brain of a non-obese animal model of T2D results in alterations of insulin signaling along with an impairment of mitochondrial activity and cognitive functions. Additionally, we describe a novel role for BVR-A by demonstrating that BVR-A functions a shuttle for GSK3β within the mitochondria and fosters mitochondrial metabolism in response to insulin. Our results also demonstrate that the loss of BVR-A is an early event during the development of brain insulin resistance in T2D, since it manifests before the accumulation of canonical hallmarks of insulin resistance, i.e., inhibited IRS1.

## 2 Materials and methods

### 2.1 GK and wistar rats

All procedures followed Swiss federal law on animal experimentation and were approved by EXPANIM-SCAV (#VD2610). Male GK and Wistar rats from Charles River (L'Arbresle, France) were housed in pairs on a 12-hr light-dark cycle with lights on at 07:00, room temperature at 21–23 °C, humidity at 55%–60 %, and with tap water and food (Kliba Nafag 3436, Provimi-Kliba, Kaiseraugst, Switzerland) provided *ad libitum*. Localized ^1^H Magnetic Resonance Spectroscopy (MRS) experiments described below were performed at 6 months of age. One-week prior MRS, a glucose tolerance test (GTT) was performed after 16 h fasting. Briefly, a 30-μL blood sample was collected from the tail tip, and serum was stored to measure fasting glucose and insulin. Then, animals were weighted and injected i.p with 2 g/kg of glucose prepared as 30 %(w/v) in saline. Glycemia was measured from tail blood immediately before and up to 4 h after glucose administration using the Ascencia Contour glucometer (Bayer, Zürich, Switzerland). Insulin was measured with an ELISA kit (#10-1250-01; Mercodia, Uppsala, Sweden). HOMA-IR (Homeostatic Model Assessment of Insulin Resistance) used to estimate insulin resistance based on fasting glucose and fasting insulin levels was calculated using the following formula: HOMA−IR=(Fasting insulin(μIU/ml) × Fasting glucose(mmol/L))/22.5 (Table 1).

**Table 1:** Diabetes phenotype of GK rats. Abbreviations: AUC, area under the curve and HOMA-IR, homeostatic model assessment.

	Glucose tolerance test after fasting 16 h					
		fasting glycemia(mM)	fasting Insulin(μg/L)	glycemia at 2h (mM)	AUC (mmol.h/L)	HOMA-IR
Wistar	mean	3,6	0,2	6,5	12,1	3,4
	SD	0,7	0,2	1,2	6,0	2,9
	n	9	9	9	9	9
GK	mean	4,5	0,2	22,4	44,9	4,5
	SD	0,8	0,1	3,7	11,0	1,8
	n	8	8	8	8	8
	p value	0,029	0,93	3,58271E-09	1,21627E-06	0,347

### 2.2 Cognitive test performed in rats

#### 2.2.1 Y-maze

One day before MRS, spontaneous alternation was observed in a Y-maze with three arms measuring 35 cm long, 9 cm wide and 30 cm height, and converging to equal angles, which was placed in a room with large visual cues on the walls. The animals were placed at the bottom of one arm of the Y-maze and allowed to explore freely all three arms for a single 8 min session in the penumbra (15 lx). The measured spontaneous alternation behavior was used to assess hippocampal-dependent spatial memory [36]. If the rat remembers the arm, it has just explored, it will therefore enter one of the other arms of the maze. Complete spontaneous alternations were defined as successive entries into the three arms and were expressed as fraction of the possible alternations in the respective test. In addition to the open field test, the number of entries in the arms of the maze also allowed to access locomotor activity and exploratory behavior of the tested rats.

#### 2.2.2 Open field

Immediately before the Y-maze test, exploratory behavior and locomotor activity were evaluated in a cubic open-field arena (50-cm wise). Rats were placed in the central area of the arena and allowed to explore it over 5 min. The number of crossings of the squares and the number of rearing movements with forepaws were recorded. Rearing with the forepaws pressed against the walls was not considered.

### 2.3 BVR-A^−/−^ mouse

All the experiments were performed in strict compliance with the Italian National Laws (DL 116/92), and the European Communities Council Directives (86/609/EEC). The experimental protocol was approved by the Italian Ministry of Health (#522/2020-PR). C57BL/6J WT mice were purchased from Charles River. BVR^−/−^ mice were kindly provided by Prof. Paul BD and the colony was bred at our animal facility at Sapienza University of Rome. BVR^−/−^ mice were generated as previously described [27]. Briefly, two LoxP sequences were inserted into the mouse germ line flanking exon 3 of Blvra for deletion via Cre recombinase. BVR^−/−^ mice were backcrossed to WT C57BL/6J mice for 14 or more generations before these studies. WT littermates were used as controls in behavioral assays. Age-matched WT C57BL/6J mice were used as controls for biochemical experiments.

### 2.4 Cognitive test performed in mice

#### 2.4.1 Novel object recognition (NOR)

The Novel Object Recognition (NOR) task is used to evaluate cognition, particularly recognition memory, in rodent models of CNS disorders. All experimental groups (Wistar, GK rats, BVR−/− and C57BL/6 mouse) were involved in the test procedures. This test is based on the spontaneous tendency of rodents to spend more time exploring a novel object than a familiar one. The task procedure consists of three phases: habituation, familiarization, and test phase. In the habituation phase at 1st day, each animal is allowed 10 min to freely exploring the open-field arena (50 cm deep × 30 cm widths × 30 cm height) in the absence of objects. During the familiarization phase on the 2nd day, a single animal is placed in the open-field arena containing two identical objects (two balls), for 10 min. To prevent coercion to explore the objects, rodents are released against the center of the opposite wall with its back to the objects. The experimental context is not drastically different during the familiarization and the test phase. In the test phase after 24 h, the animal is returned to the open-field arena with two objects, one is the familiar object and the other is novel (ball + plastic brick) [37,38]. The discrimination index and preference index percentage are recorded. Discrimination index (DI), allows discrimination between the novel (TN) and familiar (TF) objects [DI = (TN − TF)/(TN + TF)]. The preference index (PI) is a ratio of the amount of time spent exploring any one of the two objects in training phase (A, B) or the novel one in test phase (C) over the total time spent exploring both objects, i.e., A, B or C/(A, B + C) × 100 (%) in the test phase. Therefore, a preference index above 50 % indicates novel object preference, below 50 % familiar object preference, and 50 % no preference [37].

#### 2.4.2 Y-maze arm test

The Y-maze test was carried out as previously described [39].Spatial memory and exploratory activity were measured using a Y-maze apparatus, where each arm is 40 cm long, 8 cm high, 15 cm wide at the bottom, and 10 cm wide at the top. The arms (A, B and C) are all placed at 120° to each other and converge in an equilateral triangular central area that is 4 cm at its longest axis. The mice were placed at the bottom of one arm of the Y-maze and allowed to move freely through the maze for a single 5-min session. The task comprises two distinct phases: (i) the training session, also referred to as session one, and (ii) the testing session. In session one, spatial reference memory is evaluated by introducing the mouse to the Y-maze, with one arm closed off during training. Following a 30-min (Inter-trial interval), during which the mouse is removed from the maze, the (ii) testing session begins. In this phase, the mouse is reintroduced to the maze with the previously closed-off arm now accessible [39]. The evaluation involves assessing alternation or spontaneous alternation, defined as successive entries into the three arms in overlapping triplet sets, providing insights into the capacity of spatial short-term memory.

### 2.5 Localized ^1^H Magnetic Resonance Spectroscopy (MRS)

MRS was carried out in a 14.1 T magnet with a horizontal bore of 26 cm (Magnex Scientific, Abingdon, United Kingdom), equipped with a 12-cm internal diameter gradient coil insert (400 mT/m, 200μs), and interfaced to a DirectDrive console (Agilent Technologies, Palo Alto, CA, United States). Radio frequency transmission and reception were achieved with a home-built quadrature surface coil resonating at 600 MHz. Spontaneously breathing rats were anesthetized with 2–2.4 % isoflurane (Animalcare, York, UK) in a 1:1 O_2_:air mixture, and fixed in a home-built holder with a bite bar and two ear inserts. Respiration and temperature were continuously monitored using a MR-compatible system (Small Animal Instruments, Inc., Stony Brook, NY, United States). Body temperature was maintained at 37 °C by warm water circulation. Delivered isoflurane level was continuously adjusted to maintain respiration at 60–90 breaths/min. A volume of interest (VOI) of 18 μL was placed in the dorsal hippocampus (2 mm × 3 mm × 3 mm) according to anatomical landmarks in T_2_-weighted fast-spin-echo images. Field homogeneity in the VOI was achieved with FAST(EST)MAP, and spectra were acquired using SPECIAL with echo time of 2.8 ms, repetition time of 4 s, and 320 scans [40]. The concentrations of brain metabolites were determined with LCModel (Stephen Provencher Inc., Oakville, ON, Canada), including a Mac spectrum in the database and using the unsuppressed water signal measured from the same VOI as internal reference [41]. Given the high correlation between phosphorylcholine and glycerophosphorylcholine signals, total choline levels are reported. Cramér-Rao lower bounds provided by LCModel were systematically above 30 % for *scyllo*-inositol, which was thus not used for further analyses.

### 2.6 Intranasal insulin treatment (INI)

C57BL/6J and BVR-A^−/−^ mice received intranasal insulin (Humulin®R, Ely-Lilly, Inadianapolis, IN, USA) administration (2 UI total, 10 μL/nostril) or vehicle (saline) (n = 3/group) and then sacrificed after 30 min for biochemical analyses.

### 2.7 T2D subjects

For this study, we recruited eighteen consecutive T2D individuals referring to the Diabetes and Endocrinology outpatient clinics of Sapienza University, Rome, Italy, and nine age-, gender- and BMI matched healthy subjects, as a control group. All the study participants underwent complete clinical workup including medical history collection, clinical examination, anthropometric measurements and laboratory tests. Weight, height and waist circumference were measured, and body mass index calculated [BMI; weight (kg) x squared height (m^2^)]; systemic systolic (SBP) and diastolic (DBP) blood pressure were assessed after 5 min resting and mean values of three consecutive assessments were recorded. Overnight fasting blood samples were obtained in all the study participants for routine biochemistry. Fasting blood glucose (FBG, mg/dL), glycosylated hemoglobin (HbA1c, % - mmol/mol), total cholesterol (mg/dL), high-density lipoprotein cholesterol (HDL, mg/dL), triglycerides (mg/dL), were measured by centralized standard methods. Low-density lipoprotein (LDL) cholesterol value was obtained using Friedewald formula. Diabetes mellitus has been diagnosed according to the American Diabetes Association 2009 criteria [42]. Clinical and biochemical characteristics of the study population are shown in Table 2. The study was reviewed and approved by the Ethics Committee of Sapienza University of Rome (ID #3550, 26 February 2015) and was conducted in conformance with the Helsinki Declaration. Written informed consent was obtained from the subjects before participating in the study.

**Table 2:** Clinical and biochemical characteristics of T2D patients and of controls.

	T2D (n = 18)	Controls (n = 9)	*p*-value
Gender (M%)	61 %	67 %	0.92*
Age (years)	66 ± 12	63 ± 9	0.27
BMI (kg/m^2^)	28,3 ± 4.2	25,49 ± 3.7	0.18
SBP (mmHg)	139.4 ± 20.9	123.8 ± 13.4	0.03
DBP (mmHg)	81.9 ± 11.1	81.5 ± 8.6	0.94
FBG	133.6 ± 36.7	88 ± 9.8	0.001
HbA1c (%)	7.3 ± 1.1	5.2 ± 0.2	0.001
Triglycerides (mg/dl)	155 ± 72.3	94.7 ± 22.1	0.01
Total Cholesterol (mg/dl)	186.7 ± 26.4	208.5 ± 20.8	0.02
HDL (mg/dl)	45.2 ± 10.5	60 ± 14.2	0.01
LDL (mg/dl)	110.5 ± 25.7	129 ± 19.4	0.04

Values are mean ± SD for continuous variables; percentage (number) for categorical variables*.

p-value: Student t-test (continuous variables) and chi-square test* (prevalence, for categorical variables). BMI, body mass index; SBP, systolic blood pressure; DBP, diastolic blood pressure; FBG, fasting blood glucose; HbA1c, glycated haemoglobin A1c; HDL, high-density lipoprotein cholesterol; LDL, Low-density lipoprotein cholesterol.

### 2.8 Isolation of PBMC

PBMC were isolated from overnight fasting blood samples at each of the time points of the OGTT (0-30-60-90-120-180 min). ACD-A-anticoagulated blood was centrifuged at 800×*g* for 30 min and the top layer containing plasma was removed. The remaining blood was diluted with an equal volume of phosphate-buffered saline, pH 7.4 (PBS), containing 0.05 M ethylenediaminetetraacetic acid (EDTA; Invitrogen). 12.5 ml of diluted blood was layered over 25 ml of the Ficoll-Paque PLUS (GE Healthcare). Gradients were centrifuged at 400×*g* for 30 min at room temperature in a swinging-bucket rotor without the brake applied. The PBMC interface was carefully removed by pipetting and washed with PBS-EDTA by centrifugation at 250×*g* for 10 min. PBMC pellets were suspended in ammonium-chloride-potassium (ACK) lysing buffer (Invitrogen) and incubated for 10 min at room temperature with gentle mixing to lyse contaminating red blood cells (RBC), then washed with PBS-EDTA. PBMC were cryopreserved in liquid nitrogen in fetal calf serum (FCS; Invitrogen) containing 10 % dimethyl sulfoxide (DMSO; Thermo Fisher Scientific) and stored until required for downstream analyses.

### 2.9 Post-mortem AD brain

Brain tissues from well characterized subjects were provided by Sanders‐Brown Center on Aging of the University of Kentucky. All the studies were performed on the inferior parietal lobule (IPL) of non‐disease control, MCI or AD cases. Clinical diagnosis of disease stage was made as described previously [43,44]. Age and gender are listed in Table 3. The short post‐mortem interval range was between 2 and 4 h and was comparable between the three groups. The degree of cognitive impairment was assessed using the Mini Mental State Examination (MMSE) (and listed in Table 3).

**Table 3:** Autopsy case demographics. Abbreviations: MMSE, minimental state examination; PMI post-mortem interval.

Group	Age	Sex	Race	APOE	MMSE	PMI
Control	85	F	White	3/3	30	2,12
Control	92	F	White	3/3	24	1,33
Control	87	M	White	3/3	29	2,42
Control	92	M	White	3/3	30	3,75
Control	88	M	White	3/3	30	2,08
Control	84	F	White	3/3	30	2,42
Control	96	F	White	3/3	30	2,03
Control	94	M	White	3/3	29	15
MCI	88	F	White	3/3	28	3
MCI	87	M	White	3/3	27	2,75
MCI	96	F	White	3/3	27	2,42
MCI	91	M	White	3/2	28	2,33
MCI	84	M	White	3/4	24	3,5
MCI	96	F	White	3/4	28	2,25
MCI	91	M	White	3/3	30	2,83
AD	92	M	White	3/4	24	1,67
AD	86	M	White	3/4	9	3,25
AD	87	F	White	3/3	0	2,67
AD	89	F	White	4/2	24	3,17
AD	95	F	White	3/3	17	2,1
AD	90	M	White	3/4	12	3,25
AD	93	F	White	3/3	0	2,75
AD	83	M	White	NA	25	2,77

### 2.10 Cell culture and treatment

SHSY-5Y cells were grown in Dulbecco's modified Eagle's medium: Nutrient Mixture F12 (DMEM/F12; Aurogene, Rome, Italy), supplemented with 10 % fetal bovine serum (FBS; Aurogene) and 1 % penicillin (Sigma-Aldrich, St. Louis, MO, USA). Cells were maintained at 37 °C in a saturated humidity atmosphere containing 95 % air and 5 % CO2. SHSY-5Y cells were seeded in 24-wells plates (75 k/well) for 48 h before insulin treatment. To test the effects produced by silencing BVR-A, cells were seeded at density of 75 × 10^3^ in 24 wells culture dishes in DMEM F-12 with 10 % FBS, without antibiotics. Following, cells were transfected with 10 pmol of a small-interfering RNA (siRNA) for BVR-A (Ambion, Life Technologies, LuBioScience GmbH, Lucerne, Switzerland, #4392420; sense sequence GACCUGGUCUAAAACGAAAtt; antisense sequence UUUCGUUUUAGACCAGGUCct) using Lipofectamine ®RNAi MAX reagent (Invitrogen, Life Technologies, Lu-BioScience GmbH, Lucerne, Switzerland, #13778-075) according to the manufacturer's instructions and based on preliminary tests, as reported in Supplementary Fig. 8. After 48 h since siRNA administration, both Ctr and siRNA-treated cells were challenged with fresh DMEM without FBS for 2 h and then stimulated with 100 nM of insulin (HumulinR, Ely-Lilly, Inadianapolis, IN, USA) for 15, 30, 60 and 120 min. For experiments involving Lithium, cells were pre-treated with 10 mM lithium chloride (LiCl) for 24 h before insulin treatment. For siRNA-treated cells Li was added after 24 h since siRNA administration. At the end of the treatment, medium was discarded, and cells were washed twice with cold PBS, collected, and proteins were extracted as described below.

### 2.11 MitoSOX^tm^ assay red mitochondrial superoxide indicator

MitoSOX were evaluated in SHSY-5Y (Cat#EKU02723, Biomatik) according to the manufacturers’ instructions.

### 2.12 Seahorse XF analyzer respiratory assay

Cellular oxygen consumption rate (OCR) and extracellular acidification rate (ECAR) were detected using XF Cell Mito Stress Test (Agilent) measured by the extracellular flux analyzer XFe96 (Seahorse Bioscience, Houston, TX, USA) in the HypACB facility at Sapienza University. SHSY-5Y cells were cultured on XFe culture 96-wells miniplates for 24 h (10000/well) and transfected with small-interfering RNA (siRNA) for BVR-A. After 24 h the medium was supplemented with 10 mM lithium chloride (LiCl) for 24h were indicated. The sensor cartridge for XFe analyzer was hydrated in a 37 °C non-CO_2_ incubator a day before the experiment. According to the manufacturer instructions, stressors concentrations were optimized and added as follows: 1 μM oligomycin as complex V inhibitor, 1.5 μM FCCP (uncoupler agent) and 0.5 μM rotenone/antimycin A (inhibitors of complex I and III). During sensor calibration, cells were incubated in a 37 °C non-CO_2_ incubator in 180 μl assay medium (XF base medium supplemented with 10 mM glucose, 10 mM pyruvate and 2 mM L-glutamine at pH 7.4, was used to wash the cells and replace the growth medium); 120 or 30 min before Seahorse 100 nM of insulin (Humulin R, Ely-Lilly, Inadianapolis, IN, USA) was added if indicated at the following time points: 30’ and 120’.

### 2.13 Confocal microscopy and image acquisition

For immunofluorescent staining, cells were plated on coated glass coverslips. Before the use the glass coverslips were funzionalized with the following procedures: 1hr in HCL 1 M, 3 washes with milliQ, 3 washes of 30 min in EtOH 70 %. At the end of the funzionalization protocol cells were seeded in 24-wells plates (40 k/well) and then treated. At the end of treatment cells were incubated with mitotracker orange (Thermo Fisher Scientific, M7510) following manufactures’ instructions. At the end of the incubation, cells were washed three times with PBS and fixed in 4 % paraformaldehyde for 30 min. After fixation, cells were washed twice with PBS and permeabilized for 30 min with permeabilization buffer composed by 0.2 % Triton-X100 and PBS. Cells were blocked for 1 h with a solution containing 3 % normal goat serum and 0.2 % Triton X-100 in PBS and then were incubated overnight at 4 °C with following antibodies: anti-BVR-A, pGSK3β^S9^ (Table 4). Cells were washed with PBS and then incubated with Alexa Fluor plus-647 nm and −488 nm secondary antibodies (Invitrogen Corporation, Carlsbad, CA, USA) at 1:1000 for 1 h at room temperature. Cells were then washed again and incubated with DAPI solution. For each group of treatment staining was performed by omitting primary antibodies to establish nonspecific background signal. Cover slips were placed using a drop of Fluorimount (Sigma-Aldrich, St Louis, MO, USA). Images were collected on an Olympus IX73 microscope equipped with X-Light V3 spinning disk confocal imager (CrestOptics), a LDI laser source and a Prime BSI Scientific CMOS (sCMOS) camera, 6.5 μm pixels (Photometrics) with a UPlanSApo100x/1.45 oil objective. The used Z step size was 0.1 μm. All the images were acquired by using Metamorph software version 7.10.2 (Molecular Devices) and then analyzed with ImageJ software.

**Table 4:** Antibodies employed in the present study. Abbreviations: HRP, horseradish peroxidase; G, goat; M, mouse; R, rabbit.

*Antigen*	*Supplier*		*Host*	*Diluition*	*Supplier*
*Primary Antibodies:*					
IRβ	Cell signaling tecnology		M	1:1000	#3020s
phospho(Tyr1158/1162/1163)-IRβ	Genetex		R	1:1000	GTX25681
anti-IRS1	Cell signaling tecnology		R	1:1000	#3407s
phospho(Ser636)-IRS1	Gene tex		R	1:500	GTX 32400
phospho(Tyr632)-IRS1	Santa Cruz Biotechnology		R	1:1000	sc-17196
3NT	Sigma Aldrich		M	1:1000	N5538
HNE	Novus Biologicals		G	1:2000	Nb-10063093
p-Akt (Ser 473)	Cell signaling Technology		R	1:1000	#93H12
Akt	Biorad Laboratories		R	1:1000	vma00253K
GSK3β (C-terminal)	abcam	IP	M	1:100	ab93926
		Western Blot	M	1:1000	
phospho(Ser9)-GSK3β	Cell signaling tecnology	Western Blot	R	1:1000	#5558
		IF	R	1:200	
BVR-A	Sigma Aldrich	Western Blot	R	1:1000	B8437
	Santa Cruz Biotechnology	IF	M	1:100	#sc-393385
	Sigma Aldrich	IP	R	1:100	B8437
	Santa Cruz Biotechnology	Western Blot	M	1:500	#sc-393385
total OXPHOS	abcam		M	1:5000	ab110413
Complex1(NDUFB8)	Novus Biological		R	1:1000	NBP2-7558
Atf4	Santa Cruz Biotechnology		M	1:500	sc-390063
Atf5	Invitrogen		G	1:1000	PA5-17988
Grp75	Santa Cruz Biotechnology		M	1:1000	sc-133137
Chop	Invitrogen		M	1:1000	MA1-250
Sirt3	abcam		R	1:1000	ab189860
Hsp60	Santa Cruz Biotechnology		M	1:1000	sc-13115
Pgc1α	Santa Cruz Biotechnology		R	1:1000	sc-13067
Tfam	Santa Cruz Biotechnology		M	1:500	sc-166965
Gpx	Santa Cruz Biotechnology		M	1:1000	sc-133160
Catalase	Santa Cruz Biotechnology		M	1:1000	sc-271803
Sod-2	Santa Cruz Biotechnology		M	1:1000	sc-137254
*HRP-conjugated secondary antibodies:*					
mouse IgG	Bio-Rad			1: 20 000	L005662
rabbit IgG	Bio-Rad			1: 20 000	L005661
goat IgG	Sigma-Aldrich			1: 5000	A5420
mouse IgG	Rockland			1:1000	188817–33
rabbit IgG	Rockland			1:1000	188816–33
*Alkaline phospatase-conjugated secondary antibodies:*					
goat IgG	Sigma-Aldrich			1: 5000	A4187
mouse IgG	Sigma-Aldrich			1: 5000	A1293

### 2.14 Isolation of mitochondria from cells

For mitochondrial extraction, SHSY-5Y cells were washed in PBS dislodged and pelleted by centrifugation at 2700 rpm for 5 min at 4 °C. The pellet was resuspended in 700 μl of the HypoB and incubated on ice for 1 h. Cells were than homogenized using a 1 ml glass-teflon potter (100 strokes) and the walls of the potter were washed with 300 μl of HypoB (for final volume 1000 μl). The suspension was than resuspended and transferred in a 2 ml Eppendorf tube using a glass pasteur. 100 μl of HyperB were then added to the suspension in order to obtain an isotonic environment. The sample was centrifuged 1'000×*g* for 5 min at 4 °C to separate cells, debris and nuclei. The supernatant is the cytosolic fraction, marked as Cyto1. The pellet was washed with 500 μl of IsoB and centrifuged 1'000×*g* for 5 min at 4 °C to increase the yield of mitochondria. The supernatant is the cytosolic fraction, marked as Cyto2. The pellet is the nuclear fraction, marked as Nuclear. The fractions Cyto1 and Cyto 2 were pooled together and centrifuged at 20000×*g* at 4 °C for 30’. The pellet (mitochondrial fraction) was resuspended in RIPA buffer for WB analysis. The mitochondrial extraction from SHSY-5Y was performed using the following solution: Hypotonic buffer 0.1x (3.5 mM Tris HCl pH 7.8, 2.5 mM NaCl, 0.5 mM MgCl_2_), Isotonic buffer (35 mM Tris HCl pH 7.8, 25 mM NaCl, 5 mM MgCl_2_) and Hypertonic buffer 10x (350 mM Tris–HCl, pH 7.8, 250 mM NaCl, 50 mM MgCl_2_). Validation of the purity of the subcellular fractions was determined by examining Complex 1 by Western Blot analysis.

### 2.15 Samples preparation

Total protein extracts were prepared in RIPA buffer (pH = 7.4) containing 50 mM Tris-HCl (pH = 8), 150 mM NaCl, 1 % NP-40, 0.5 % sodium deoxycholate,1 mM EDTA, 0.1 % SDS, together with phosphatase and protease inhibitor (539132, Millipore, 1:100; P0044; Sigma-Aldrich, St. Louis, MO, USA; 1:100). Samples were sonicated on ice and then centrifuged at 14 000 rpm at 4 °C for 30 min to remove cellular debris. Supernatants were collected to determine total protein concentrations by the BCA method (Pierce, Rockford, IL, USA).

### 2.16 Western blot

Ten μg of proteins were separated by 4–15 % gradient sodium dodecyl sulfate–polyacrylamide gel electrophoresis (SDS-PAGE), using Criterion TGX (Tris-Glycine extended) Stain-Free precast gels (Bio-Rad, Hercules, CA, USA) in Tris/Glycine/SDS (TGS) Running Buffer (Bio-Rad Laboratories, # 1610772). All the samples were loaded on the same gel by using 26-well gel (Bio-Rad Laboratories, # 5678085). Immediately after electrophoresis, the gel was then placed on a Chemi/UV/Stain-Free tray and then placed into a ChemiDoc MP imaging System (Bio-Rad Laboratories, # 17001402) and UV-activated based on the appropriate settings with Image Lab Software (Bio-Rad Laboratories) to collect total protein load image. Following electrophoresis and gel imaging, the proteins were transferred via the TransBlot Turbo semi-dry blotting apparatus (Bio- Rad Laboratories, # 1704150) onto nitrocellulose membranes (Bio-Rad, Hercules, CA, USA, # 162–0115). Membranes were blocked with 3 % of bovine serum albumin (SERVA Electrophoresis GmbH, Heidelberg, Germany) in TBS solution containing 0.01 % Tween 20 and incubated over night at 4 °C with the following primary antibodies listed in Table 4. Subsequently, membranes were incubated at room temperature with the respective horseradish peroxidase-conjugated secondary antibodies for 1 h listed in Table 4. Membranes were developed with Clarity enhanced chemiluminescence (ECL) substrate (Bio-Rad Laboratories, Hercules, CA, USA, #1705061) and then acquired with ChemiDoc MP (Bio-Rad, Hercules, CA, USA) and analyzed using Image Lab 6.1 software (Bio-Rad, Hercules, CA, USA) that allows the normalization of a specific protein signal by the total protein load. Total protein staining measures the aggregate protein signal (sum) in each lane and eliminates the error that can be introduced by a single internal control protein. Total protein staining is a reliable and widely applicable strategy for quantitative immunoblotting. It directly monitors and compares the aggregate amount of sample protein in each lane, rather than using an internal reference protein as a surrogate marker of sample concentration. This direct, straightforward approach to protein quantification may increase the accuracy of normalization. Total load can be detected by taking advantage of the stain-free technology (Bio-Rad, Hercules, CA, USA). Stain-free imaging technology utilizes a proprietary trihalo compound to enhance natural protein fluorescence by covalently binding to tryptophan residues with a brief UV activation. Images of the gel or membrane after transfer can easily be captured multiple times without staining and destaining steps.

### 2.17 Slot blot analysis

To evaluate total protein-bound (i) 4-hydroxy-2-nonenal (HNE) and (ii) 3-nitrotyrosine (3-NT) levels, 3 μL of hippocampal proteins homogenate were incubated with 6 μL of Laemmli Buffer (0.125 M Tris base pH = 6.8, 4 % (v/v) SDS and 20 % (v/v) glycerol) for 20 min at room temperature and then loaded onto nitrocellulose membrane as described below. For Protein carbonyls 3 μL of hippocampal proteins homogenate was derivatized at room temperature for 20 min in 10 mM DNPH and 5 μL of 12 % sodium dodecyl sulfate (SDS). Samples were than neutralized with 7.5 μL of neutralization solution (2 M tris in 30 % glycerol). Proteins (250 ng) were loaded in each well on a nitrocellulose membrane under vacuum using a slot blot apparatus. Membranes were blocked for 1 h at room temperature with 3 % of bovine serum albumin (SERVA Electrophoresis GmbH, Heidelberg, Germany) in Tris-buffered saline (TBS) solution containing 0.01 % Tween 20 and incubated at room temperature for 2 h with the following primary antibodies: anti HNE polyclonal antibody or an anti 3-NT polyclonal antibody (Table 4). Then, membranes were washed three times with TBS solution containing 0.01 % Tween 20 and incubated for 1 h at room temperature with the respective alkaline phosphatase secondary antibodies listed in Table 4. Then, the membranes were washed three times in TBS solution containing 0.01 % Tween 20 and developed with Sigma Fast BCIP/NBT (5-Bromo-4-chloro-3-indolyl phosphate/Nitro blue tetrazolium substrate). Blots were dried, acquired with Chemi-Doc MP imaging system Bio-Rad Laboratories, # 17001402) (Bio-Rad, Hercules, CA, USA) and analyzed using Image Lab 6.0 software (Bio-Rad, Hercules, CA, USA). No non-specific binding of antibody to the membrane was observed.

### 2.18 Immunoprecipitation

Magnetic beads were used to immunoprecipitate GSK3β in rats and mouse; and BVR-A in SHSY-5Y cells line (SureBeads™ Protein G Magnetic Beads; cat #161–4023, Bio-Rad Laboratories) according to manufacturer instructions. Briefly, 100 μg of proteins’ extracts were incubated over-night at 4 °C with anti-GSK3β or BVR-A primary antibody in PBS containing inhibitors (Table 4). The day after, each sample was incubated for 2 h with 100 μL of magnetic beads previously magnetized using a specific tube magnetic rack and washed three times with 1X PBS containing 0.1 % Tween20. Subsequently, the beads were recovered through the magnetic rack and washed 3 times with 1X PBS containing 0.1 % Tween20 to wash the unbound protein fractions. Then 20ul of elution buffer (Laemmli Buffer #1610737) was added to the beads and incubate for 10 min at 70°, magnetized and then the purified target protein was collected. Each sample was then subjected to Western blot as described above. Membranes were incubated overnight at 4 °C with the following primary antibodies: anti-Akt, anti-GSK3β and anti-BVR-A (Table 4) and detected by an anti-rabbit or an anti-mouse TrueBlot Secondary antibody (HRP). TrueBlot® secondary antibodies eliminate interference from the denatured/reduced heavy and light chains of the IP antibody by detecting only the native, non-reduced form of IgG. IP results were normalized on the total amount of IPed GSK3β (rats and mouse IP) or BVR-A (SHSY-5Y IP).

### 2.19 Respiratory Chain Complexes Activity and ATP content

Respiratory Chain Complexes Activity and ATP Content was performed in cryopreserved hippocampus from GK, Wistar rats and WT, BVR-A^−/−^. For the cryopreservation of brain tissue, dissections were carried out in ice-cold solution consisting of 50 mM K-Mes (pH 7.1), 3 mM K2HPO4, 9.5 mM MgCl2, and 3 mM ATP. Brain samples were then transferred into a cryotube containing 500 μl of the same solution plus 20 % glycerol and 10 mg/ml fatty acid-free bovine serum albumin (BSA). Samples were then frozen in liquid isopentane to achieve a rapid and uniform freezing and were maintained at −30 to −35 °C. Subsequently all samples were stored at −80 °C or stored in dry ice. For mitochondria isolation, tubes containing cryopreserved tissues were placed on ice, and when the cryopreservation solution was almost completely thawed, brain tissues were immediately transferred and washed in ice-cold respiratory medium consisting of 210 mM mannitol, 70 mM sucrose, 3 mM MgCl2, 20 mM Tris– HCl, and 5 mM KH2PO4/K2HPO4 (pH 7.4) plus 2 mg/ml BSA. The polarographic oxygen measurements were performed using an oxygraph-2k high-resolution respirometer (Oxygraph-2k, Oroboros instruments) with a 1.5 mL chamber equipped with a Clark oxygen electrode. The assays were carried out at a constant temperature of 37 °C and stirrer speed 750 rpm in 3 mM MgCl_2_, 20 mM taurine, 10 mM KH_2_PO_4_, 20 mM HEPES, 110 mM Sucrose, 0.1 % BSA, 5.5 mM glucose. Oxygen flux (*J*O_2_), which is directly proportional to oxygen consumption rate, was continuously recorded with a 1 s sampling rate using DatLab software (Oroboros Instruments, Austria). For measurements, the following substrates and inhibitors were used: 1 mM NADH (complex I reductant), 5 mM succinate (complex II reductant), 0,5 μM rotenone (complex I inhibitor), 2.5 μM antimycin A (complex II inhibitor), 2 mM ascorbate and 0.8 mM tetramethylene phenylene diamine (TMPD) reductants of complex IV, 2 mM NaCN (inhibitor of complex IV).

### 2.20 Subcellular fractionation

Subcellular fractionation used to isolate cytosolic, mitochondrial and nuclear fractions from rats and mice hippocampal samples, was performed according to Dimauro et al. [45].Validation of the purity of the subcellular fractions was determined by examining Complex I in the mitochondrial fraction and Polymerase II for nuclear extraction by Western Blot analysis.

### 2.21 Proteomic analyses

An in-depth label free proteomics investigation has been carried out on protein fractions derived from hippocampal samples of both experimental groups, Wistar *vs* GK, and WT *vs* BVR-A^−/−^. Protein Extracts (50 μg) resulting from each condition (1st dataset: 8 GK *vs* 8 Wistar rats; 2nd dataset: 4 BVR-A^−/−^ *vs* 4 WT) have been handled performing the protocol for enzymatic digestion with Filter-Aided Sample Preparation (FASP), as described by Distler et al. [46].Briefly, reduction (DTT 8 mM), alkylation (IAA 50 mM) and digestion by trypsin (final trypsin concentration of 0.01 μg/μL; 1: 50 w/w ratio with respect to the protein content) were performed on filter tubes (Nanosep centrifugal device with Omega membrane-10 K MWCO). Tryptic peptides from each digested sample were analyzed by a Orbitrap Fusion Lumos Tribrid (Thermo Fischer Scientific) Mass Spectrometer coupled to Ultimate 3000 RSLC nano-HPLC (Thermo Fisher Scientific) and Nanospray Flex source (Thermo Fisher Scientific). Dried peptides were loaded into PepMap RSLC C18 column (2 μm, 100 Å, 50 μm × 15 Cm; Thermo Fisher Scientific). Analyses were performed using Eluent A (Formic Acid, FA 0.1 %, v/v) and eluent B (ACN/FA; 99.9:0.1, v/v) in the following gradient elution: (i) 5 % of eluent B (7 min); (ii) from 5 to 35 % of eluent B (113 min); (iii) from 35 to 99 % of B (15 min); (iv) 99 % of B (10 min); (v) from 99 to 5 % of B (2 min); (vi) 5 % of B for column conditioning (13 min). The column was kept at 40 °C and operated at a flow rate of 300 nL/min; the injection volume was set at 5.0 μL. Peptides were then eluted into The Orbitrap Fusion Lumos Tribrid (Thermo Fisher Scientific, Waltham, MA, USA). Tandem mass spectrometry analysis were performed in positive full-scan acquisition mode in the 350–2000 *m/z* range working at higher energy- Collision Dissociation (HCD) cell and in Data Dependent Acquisition (DDA) mode. Each sample was analyzed in 3 technical replicates.

#### 2.21.1 Label free quantification (LFQ)

Raw data collected from LC/MS analysis of both experimental datasets have been processed by PEAKS software (PEAKS Studio v. XPRO) for both qualitative and quantification analysis. According to the experimental groups, qualitative protein identification was obtained using the PEAKS algorithm by searching against *Rattus Norvegicus* UniProt KB/Swiss-Prot taxonomy (for GK dataset) and *Mus musculus* UniProt KB/Swiss-Prot taxonomy (for BVR-A^−/−^ dataset). Search parameters were set as follows: 15.0 ppm as automatic parent mass error tolerance, 0.02 Da as fragment mass error tolerance; minimum two peptides matched per protein; one missed cleavage: carbamydomethylation of cysteines and oxidation of methionines as fixed and variable modifications respectively; false positive rate (FPR) of the identification algorithm lower than 1 % and 0.1 % at protein and peptide level respectively. Likewise, protein quantification was performed using PEAKS software. In particular Label Free Quantification (LFQ) (GK *vs* Wistar rats; BVR-A^−/−^ *vs* WT) was developed considering technical replicates available for each experimental condition following the hypothesis that each group was an independent variable. The method is based on the relative abundance of all peptide features detected in at least two samples. Peptide feature detection is performed independently on each sample, allowing for the detection of overlapping features. Fold change of regulation was set higher than ≥20 %; only modulated proteins with p-Value <0,05 were considered significant.

#### 2.21.2 Protein ontologies and network analysis

To identify biologically relevant molecular pathways, Proteomic datasets were analyzed with the use of QIAGEN IPA (QIAGEN Inc., https://digitalinsights.qiagen.com/IPA). Functional associations and relevant networks have been explored. The analysis parameters were set as the following: direct and indirect relationships, endogenous chemical substances included all molecules and/or relationships considered as the summary filter. The most significant categories associated with the loaded datasets were identified by calculating their significance (p-value, Fischer test). A p-value threshold was set at 0.05, which showed the probability of association between genes/proteins present in the datasets and each pathway (canonical pathway, and biological function).

### 2.22 Statistical analysis

Statistical analyses were performed using Student *t*-test for the evaluation of differences between two groups. One-way analyses of variance (ANOVA) with specific post-hoc test was used for the valuation of differences between more than two groups. Associations were tested by Pearson's coefficient. Details about each test is reported in figure legend. All statistical analyses were performed using Graph Pad Prism 9.0 software (GraphPad, La Jolla, CA, USA).

## 3 Results

### 3.1 Altered insulin signalling is associated with impaired mitochondrial metabolism in the hippocampus of GK rats

**Fig. 1:**
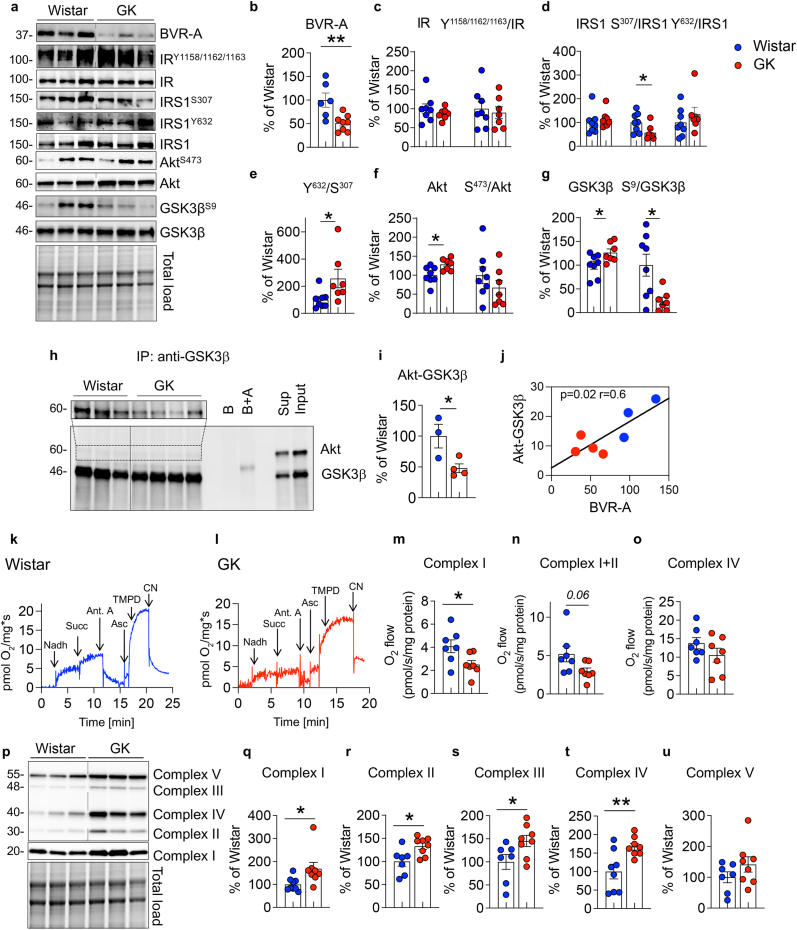
Brain insulin signaling alterations are associated with an impaired mitochondrial metabolism in GK rats. Changes of BVR-A and of the insulin signaling pathway's proteins levels, i.e., IR, IRS1, Akt and GSK3β were evaluated in the hippocampus of Wistar (n = 6–9 independent samples) and GK (n = 7–8 independent samples) rats. **(a)** Representative Western blot images and densitometric evaluation of **(b)** BVR-A protein levels (p = 0.006, GK vs Wistar); **(c)** IR protein levels (p = 0.38, GK vs Wistar), and IR activation (evaluated as pIR^Y1158/1162/1163^/IR ratio; p = 0.83, GK vs Wistar); **(d)** IRS1 protein levels (p = 0.51, GK vs Wistar), IRS1 inhibition (evaluated as S^307^/IRS1 ratio; p = 0.04, GK vs Wistar) and IRS1 activation (evaluated as Y^632^/IRS1 ratio; p = 0.35, GK vs Wistar); **(e)** IRS1 activation state index (evaluated as IRS1 Y^632^/S^636^ ratio; p = 0.03, GK vs Wistar); **(f)** Akt protein levels (p = 0.01, GK vs Wistar), and Akt activation (evaluated as S^473^/Akt ratio; p = 0.12, GK vs Wistar); **(g)** GSK3β protein levels (p = 0.03 GK vs Wistar) and GSK3β inhibition (evaluated as S^9^/GSK3β ratio; p = 0.01 GK vs Wistar). (**h)** Representative Western blot images and **(i)** densitometric evaluation of the Akt/GSK3β complex isolated through the immunoprecipitation assay from the hippocampus of Wistar (n = 3 independent samples) and GK (n = 4 independent samples) rats; p = 0.03 GK vs Wistar. Lanes description: lanes 1 to 3: Wistar; lanes 4 to 7: GK; Lane 8: empty; lane 9: beads alone (B); lane 10; beads plus the anti-GSK3β primary antibody, but no sample (B+A); lane 11: empty; lane 12: supernatant collected following beads magnetization; and lane 13: Input. **(j)** A significant association was found between the Akt/GSK3β complex, and the respective BVR-A protein levels evaluated in the same samples (Pearson r = 0.6; p = 0.02). **(k**–**o)** Representative traces showing oxygen consumption rates (OCR) in Wistar and GK rats. Arrows indicate the addition of substrates and/or inhibitors (1 mM NADH, 5 mM Succinate, 2.6 μM Antymicin, 2.13 mM Ascorbate, 0.8 mM N,N,N′,N′-Tetramethyl-p-phenylenediamine (TMPD) and 1 mM Sodium Cyanide (CN). Quantification of the OCR, normalized for proteins content (pmol O_2_/s/mg proteins) after the addition of **(m)** NADH (Complex I, p = 0.03 GK vs Wistar); **(n)** Succinate (Complex I+II, p = 0.06 GK vs Wistar); **(o)** Antimycin to block Complex III, Ascorbate and TMPD (Complex IV, p = 0.2 GK vs Wistar). **(p)** Representative Western blot images and densitometric evaluation of **(q)** Complex I (subunit NDUFB8, p = 0,02 GK vs Wistar), **(r)** Complex II (subunit SDHB, p = 0.01 GK vs Wistar), **(s)** Complex III (subunit UQCRC2, p = 0.04 GK vs Wistar), **(t)** Complex IV (subunit MTCO1, p = 0.007 GK vs Wistar), and **(u)** Complex V (subunit ATP5A, p = 0.2 GK vs Wistar). The densitometric values are given as percentage of Wistar set as 100 %. Data are presented as means ± SEM. Statistical significance was determined using Student t-test analysis (*p < 0.05, **p < 0.01).

To understand the molecular alterations induced in the brain by the development of insulin-resistance and T2D, we evaluated changes in BVR-A and the insulin signaling pathway by the mean of IR, IRS1, Akt and GSK3β protein levels and phosphorylation, in the hippocampus of GK rats as compared to Wistar rats (Fig. 1a). BVR-A levels are significantly reduced in GK rats' hippocampus (Fig. 1b) and BVR-A reduction strongly associates with the HOMA-IR (an index of insulin resistance) (Table 1, Pearson r = −0.54, p = 0.04). No significant differences in either IR protein levels or activation state (Y^1158/1162/1163^/IR) were observed between the 2 groups (Fig. 1c). Rather, IRS1 activation [evaluated as ratio between the two main activation/inhibition phosphorylation sites, Y^632^/S^307^] was significantly elevated in GK rats and was mainly driven by a significant reduction of IRS1 inhibition (S^307^ phosphorylation) (Fig. 1d and e). These findings are consistent with the proposed role for BVR-A as a key element in the regulatory loop formed by IR, IRS1 and BVR-A upstream in the signaling aimed at preventing IRS1 excessive activation in response to insulin [47]. Despite IRS1 hyper-activation, no changes were observed for Akt activation (S^473^/Akt) (Fig. 1f), suggesting the uncoupling of the insulin signaling at this level. To further support the lack of Akt activation we analyzed the activation of GSK3β, one of the main targets of Akt. Under physiological conditions, Akt-mediated inhibition (S^9^ phosphorylation) of GSK3β, which activates glycogen synthesis and represents a neuro-protective and pro-survival mechanism, is rapidly induced in response to insulin [48]. Intriguingly, GSK3β inhibition (S^9^/GSK3β) was significantly reduced in GK rats as compared to Wistar (Fig. 1g). The observation that S^9^/GSK3β was diminished to a greater extent than that observed for S^473^/Akt, indicated that inhibition of GSK3β by Akt was suboptimal in GK rats. As previously demonstrated, BVR-A works as a scaffold protein [19,24] and facilitates the binding of Akt to GSK3β, leading to GSK3β inhibition [23,24]. Thus, we hypothesized that reduced BVR-A protein levels might be responsible for the reduced GSK3β inhibition in the hippocampi of GK rats. To test this possibility, we immunoprecipitated GSK3β and analyzed the formation of the Akt/GSK3β complex in Wistar and GK rats' hippocampal samples. Our results clearly demonstrated a significant decrease in complex formation in GK rats where reduced BVR-A levels were observed (Fig. 1h–j). Defects in the insulin signaling pathway are closely associated with impaired mitochondrial activity and brain energy metabolism [49], and it has been reported that inactive GSK3β (S^9^ phosphorylated form) promotes mitochondria bioenergetics and brain energy metabolism [50]. Hence, we evaluated mitochondrial activity in rats’ hippocampal samples by analyzing the oxygen consumption rate (OCR) measured through the Oroboros (Fig. 1k,l). Remarkably, we found a significant reduction of Complex I activity (Fig. 1m) along with a nearly significant reduction of Complex I+II (Fig. 1n) in GK rats compared to Wistar rats. No changes were observed for Complex IV (Fig. o). To understand whether the suboptimal mitochondrial activity was caused by alterations of the electron chain complexes we evaluated complexes protein levels. Surprisingly, a significant increase of Complex I (subunit NDUFB8), Complex II (subunit SDHB), Complex III (subunit UQCRC2) and Complex IV (subunit MTCO1) was observed in the hippocampus of GK rats (Fig. 1 q-t). No significant differences for Complex V (subunit ATP5A) were observed (Fig. 1u). These results suggest that an adaptive response has been activated in the brain of GK rats, wherein reduced mitochondrial activity appears to be compensated by an increase in OXPHOS chain complexes levels. However, this compensation appears to be unsuccessful. To further explore the potential role of insulin signaling and the reduced inhibition of GSK3β in the observed impairment in Complex I and Complex II activities, we performed correlation analyses that might support our hypothesis. We found that Complex I activity was significantly associated with GSK3β inhibition (Complex I activity *vs* pGSK3β^S9^: Pearson r = 0.55, p = 0.04; Complex I activity *vs* pGSK3β^S9^/GSK3β: Pearson r = 0.54, p = 0.04). These data strengthen the idea that the disruption of Akt-mediated inhibition of GSK3β might be driving mitochondrial dysfunction in the hippocampus of GK rats.

### 3.2 UPRmt is activated as an adaptive response to face alterations of insulin signaling-mitochondria axis in the brain

**Fig. 2:**
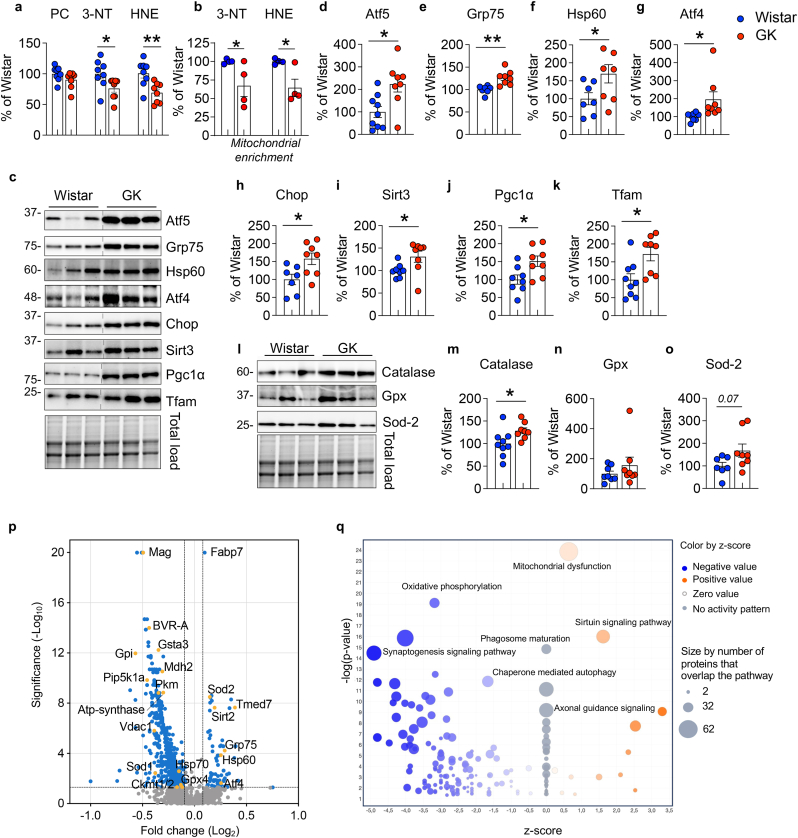
UPRmt is activated as an adaptive response to face alterations of insulin signaling-mitochondria axis in the brain. **(a)** Densitometric evaluation of markers, i.e., protein carbonyls (PC) (p = 0.15, GK vs Wistar), 3-nitrotyrosine (3-NT) (p = 0.03, GK vs Wistar) and proteins-bound 4-hydroxyl-2-nonenals (HNE) (p = 0.0051, GK vs Wistar) evaluated in the hippocampus of Wistar (n = 8 independent samples) and GK (n = 8 independent samples) rats. **(b)** Densitometric evaluation of 4-HNE (p = 0.03, GK vs Wistar) and 3-NT (p = 0.02, GK vs Wistar) evaluated in hippocampal mitochondrial extracts isolated from Wistar (n = 4 independent samples) and GK (n = 4 independent samples) rats. Changes of the Unfolded Protein Response (UPRmt) proteins [Atf5, Grp75, Hsp60, Atf4, Chop, Sirt3, Pgc1α and Tfam] and of the antioxidant enzymes [Catalase, Gpx and Sod-2] were evaluated in the hippocampus of Wistar (n = 8–9 independent samples) and GK (n = 7–8 independent samples) rats. **(c)** Representative Western blot images and densitometric evaluation of **(d)** Atf5 (p = 0.01, GK vs Wistar), **(e)** Grp75 (p = 0.002, GK vs Wistar), **(f)** Hsp60 (p = 0.04, GK vs Wistar), **(g)** Atf4 (p = 0.04, GK vs Wistar), **(h)** Chop (p = 0.04, GK vs Wistar), **(i)** Sirt3 (p = 0.03, GK vs Wistar), **(j)** Pgc1α (p = 0.01, GK vs Wistar) and (k) Tfam (p = 0.01, GK vs Wistar). **(l)** Representative Western blot images and densitometric evaluation of **(m)** Catalase (p = 0.03, GK vs Wistar) **(n)** Gpx (p = 0.33, GK vs Wistar) and **(o)** Sod-2 (p = 0.07, GK vs Wistar). Values are given as percentage of Wistar set as 100 %. Data are presented as means ± SEM. Statistical significance was determined using Student t-test analysis (*p < 0.05, **p < 0.01) **(p)** Wistar and GK hippocampal samples were tested by label-free quantitative proteomics approach and analyzed through the Ingenuity Pathway Analysis (IPA) software (QIAGEN IPA). Mass spec identified 3461 proteins and isoforms listed in Supplementary material. Volcano plot indicating differentially expressed proteins in Wistar and GK rats. Proteins showing a fold-change significantly (p < 0.05) reduced or elevated in GK vs Wistar rats are shown in blue. Highlighted in yellow proteins relevant for the current study. **(q)** Bubble diagram of Canonical Pathways across the entire dataset of proteins. The colors indicate the z-score (see legend at top right), while the size of the bubbles increases with the number of overlapping proteins. The IPA analysis display a negative regulation of pathways associated with Oxidative phosphorylation, synaptogenesis signaling pathway and chaperone mediated autophagy. A positive regulation of pathways related to mitochondrial dysfunctions and sirtuin signaling and axonal guidance signalling was observed. (For interpretation of the references to color in this figure legend, the reader is referred to the Web version of this article.)

Mitochondrial dysfunction is intimately linked to elevated oxidative stress levels in the brain, which causes oxidative damage to proteins and lipids, which affects synaptic components crucial for neuronal plasticity, cytoskeletal dynamics, and cellular communication [1]. It also results in the loss of synapses and ultimately leads to neurodegeneration [1]. Hence, we assessed the levels of well-known markers of oxidative stress damage, i.e., protein carbonyls (PC), proteins-bound 4-hydroxyl-2-nonenal (HNE), and 3-nitrotyrosine (3-NT). A decrease in 3-NT and HNE levels in hippocampal homogenates were observed (Fig. 2a). No changes were found for PC (Fig. 2a). Likewise, there was a significant reduction of both markers in mitochondrial extracts from GK compared to Wistar rats, contrary to our expectations (Fig. 2b). Given the impaired mitochondrial activity in GK rats, we anticipated an increase in the levels of oxidative stress markers. Furthermore, it's worth noting that changes in insulin signaling, reduced BVR-A levels, and reduced GSK3β inhibition are all events previously described as triggers of oxidative damage [24]. Therefore, to unravel the molecular mechanism(s) responsible for this adaptive response, where reduced mitochondrial activity coexists with reduced oxidative damage, we directed our attention to the mitochondrial unfolded protein response (UPRmt). This protective pathway is coordinated by mitochondrial chaperones and proteases and serves to restore perturbed mitochondrial proteostasis, reduces oxidative stress levels and rescue mitochondrial fitness [51]. Intriguingly, we found that levels of proteins known to promote the activation of UPRmt, i.e., Atf5, Grp75, Hsp60, Atf4, Chop (the canonical axis [51]), and Sirt3, Pgc1, Tfam (the sirtuins axis [51]) were all significantly upregulated in the hippocampus of GK compared to WT rats (Fig. d–k). We also detected a significant increase of phosphorylated eif2α, required for the activation of the UPRmt (Supplementary Fig. 1b) [51]. We did not observe any significant changes in Grp78 protein levels (Supplementary Fig. 1c) between Wistar and GK rats suggesting that this protective response is selectively mediated by the UPRmt. The activation of UPRmt parallel with the activation of the antioxidant response, primarily due to a significant up-regulation of Catalase protein (Fig. 2m). There's also a nearly significant increase in Sod-2 protein levels, while no change was observed for glutathione peroxidase (Gpx) (Fig. 2n-o). This aligns with the established role of the UPRmt in promoting the expression of Catalase and Sod-2 [52]. Moreover, it provides a possible explanation for the observed reduction in oxidative stress. GK vs Wistar hippocampal samples were also tested by label-free based quantitative proteomics approach and analyzed through the Ingenuity Pathway Analysis (IPA) software (QIAGEN). Mass spec analysis identified 3461 proteins and isoforms (Supplementary Table 1). The Volcano plot highlights BVR-A as one of the most significantly downregulated proteins in the hippocampus of GK rats (Fig. 2p). A similar pattern was observed for key proteins associated with cell energy metabolism, i.e., Gsta3, Mdh2, Pkm, Pip5k1a, Gpi, Ckm and Vdac. Conversely, proteins associated with the UPRmt, such as Grp75, Hsp60, Sirt2, and Sod2, exhibit substantial upregulation (Fig. 2p). The IPA analysis concerning significant Canonical Pathways (Supplementary Table 1) across the entire dataset of proteins found differentially expressed, display a negative regulation of pathways associated with glycolysis, mitochondrial metabolism, proteostasis network, neuronal architecture, and neuronal plasticity mechanisms. Conversely, a positive regulation of pathways related to mitochondrial dysfunctions and sirtuin signaling was observed (Fig. 2q). In line with the reduction of mitochondrial proteins levels identified with IPA analysis among which the mitochondrial Ckm, metabolite profiling *in vivo* by ^1^H MRS revealed a reduction in phosphocreatine levels in the hippocampus of GK *versus* Wistar rats (Supplementary Fig. 1 e). Moreover, we observed impaired hippocampal-dependent spatial memory, as evidenced by decreased spontaneous alternations in the Y-maze task (Supplementary Fig. 1 f). No significant changes were observed in exploratory or locomotor functions (Supplementary Fig. 1 g-i).

Overall, these observations suggest that during the development of T2D, the impairment of brain insulin signaling, and the alterations of cell energy metabolism led to the activation of neuroprotective and adaptive responses like UPRmt, aimed at repairing and preserving damaged mitochondria. This is likely required to sustain energy production for neuronal function and plasticity mechanisms, that otherwise would be severely compromised.

### 3.3 Loss of BVR-A links alterations of brain insulin signaling and mitochondrial dysfunctions

**Fig. 3:**
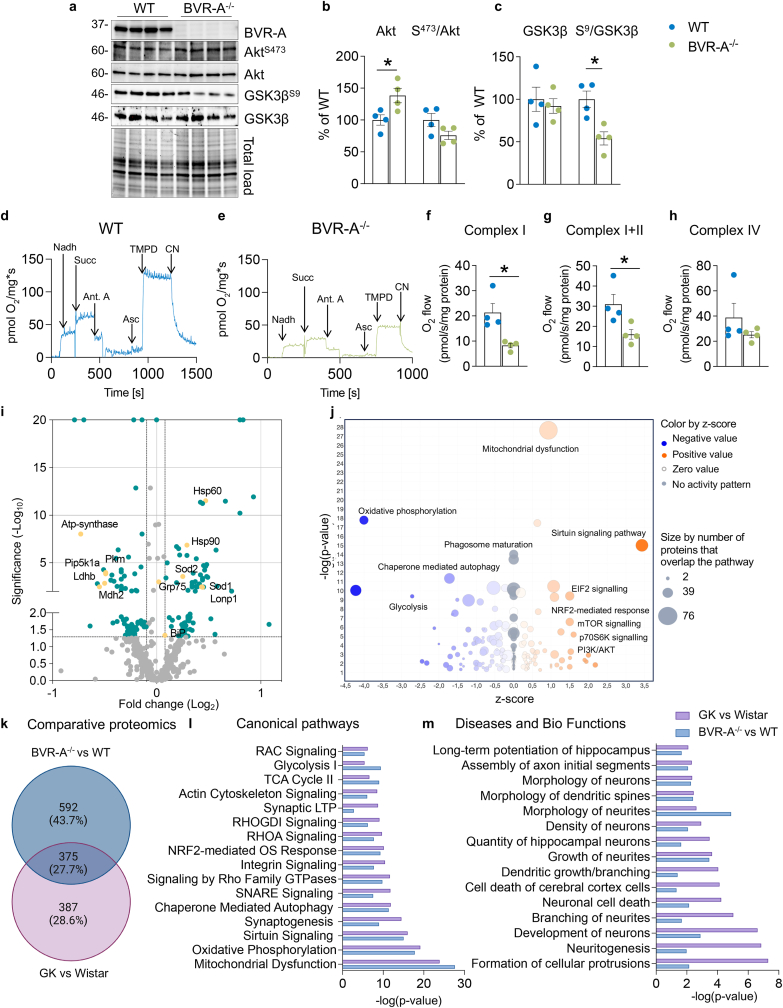
Loss of BVR-A links alterations of brain insulin signaling and mitochondrial dysfunctions. Changes of Akt and GSK3β activation were evaluated in the hippocampus of WT (n = 4 independent samples) and BVR-A^−/−^ (n = 4 independent samples) mice. **(a)** Representative Western blot images and densitometric evaluation of **(b)** Akt protein levels (p = 0.03, WT vs BVR-A^−/−^) and Akt activation (evaluated as S^473^/Akt ratio; p = 0.09, WT vs BVR-A^−/−^), **(c)** GSK3β protein levels (p = 0.65, WT vs BVR-A^−/−^) and GSK3β inhibition (evaluated as S^9^/GSK3β ratio; p = 0.01, WT vs BVR-A^−/−^). **(d and e)** Representative traces showing oxygen consumption rates (OCR) in WT and BVR-A^−/−^ mice samples normalized for proteins content. Arrows indicate the addition of substrates and/or inhibitors (1 mM NADH, 5 mM Succinate, 2,6uM Antymicin, 2,13 mM Ascorbate, 0,8 mM N,N,N′,N′-Tetramethyl-p-phenylenediamine (TMPD) and 1 mM Sodium Cyanide (CN). Quantification of OCR normalized for protein content (pmol O_2_/s/mg proteins) after the addition of **(f)** NADH (Complex I, p = 0.01, WT vs BVR-A^−/−^), **(g)** Succinate (Complex I+II, p = 0.03, WT vs BVR-A^−/−^), **(h)** Antimycin to block Complex III, Ascorbate, and TMPD (Complex IV, p = 0.2, WT vs BVR-A^−/−^). Values are given as percentage of WT set as 100 %. Data are presented as means ± SEM. Statistical significance was determined using Student t-test (*p < 0.05). **(i)** WT and BVR-A^−/−^ hippocampal samples were tested by label-free quantitative proteomics approach and analyzed through the Ingenuity Pathway Analysis (IPA) software (QIAGEN). Mass spec analysis identified 989 proteins and isoforms listed in Supplementary material. **(i)** Volcano plot indicating differentially expressed proteins in WT and BVR-A^−/−^ mice. Proteins showing a fold-change significantly (p < 0.05) reduced or elevated in WT and BVR-A^−/−^ mice are shown in green. Highlighted in yellow proteins relevant for the current study. **(j)** Bubble diagram of Canonical Pathways across the entire dataset of proteins. The colors indicate the z-score (see legend at top right), while the size of the bubbles increases with the number of overlapping proteins. The IPA analysis displays a negative regulation of pathways associated with oxidative phosphorylation, glycolysis and chaperone mediated autophagy. A positive regulation of pathways related to mitochondrial dysfunctions, sirtuin signaling, EIF-2 signalling, NRF2- mediated response, mTOR, p70S6K and PI3K/Akt signalling was observed. **(k)** Venn diagram illustrating the outcomes of a comparative analysis conducted on label-free proteomic datasets obtained from GK vs Wistar rats and BVR-A^−/−^ vs WT mice. The analysis highlights a shared set of 375 proteins exhibiting a consistent pattern of alteration across both groups. This subset constitutes 27.7 % of the total identified proteins. **(i)** Comparative analysis between the Canonical pathways identified by IPA in rats (violet) and mice (blue) groups of analysis. Data show the common alteration of mitochondria, sirtuin signaling, synaptogenesis, among others. **(m)** Biological outcomes identified by IPA analysis in Wistar/GK rats (violet) and WT/BVR-A^−/−^ (blue) groups. Data show the common alteration of long-term potentiation of hippocampus, assembly of axon initial segments and morphology of neurons, among others. (For interpretation of the references to color in this figure legend, the reader is referred to the Web version of this article.)

To emphasize the role for BVR-A in the observed alterations, we performed similar analyses in the hippocampus of a knock-out mouse model for BVR-A (BVR-A^−/−^). We observed a significant reduction of IR activation along with a consistent increase of IRS1 inhibition, demonstrated by elevated S^307^/IRS1 ratio (Supplementary Figs. 2b and c). These findings support the development of brain insulin resistance in the hippocampus of BVR-A^−/−^ mice. Downstream in the pathway we observed that Akt activation (S^473^/Akt) did not show significant changes, while a consistent reduction of GSK3β inactivation was observed in BVR-A^−/−^ when compared to WT mice (Fig. 3b and c). These observations strengthen the idea that loss of BVR-A impairs the Akt-mediated inhibition of GSK3β in agreement with data collected in GK rats and with previous studies [24]. The reduction of GSK3β^S9^ phosphorylation parallels the impairment of mitochondrial activity, as highlighted by the significant reduction in Complex I (Fig. 3f) and Complex I+II (Fig. 3g) activities reported in BVR-A^−/−^ when compared to WT mice. No significant changes were observed for mitochondrial OXPHOS complexes levels (Supplementary Figs. 2d–i). An increase of oxidative stress levels in BVR-A^−/−^ mice was also observed, as demonstrated by the accumulation of PC and 3-NT modifications (Supplementary Fig. 2j). We also measured the aforementioned UPRmt markers in this model. We found that proteins belonging to the canonical axis of the UPRmt were mainly upregulated. These include Hsp60, Chop, and the master regulator of UPRmt, Grp75, which showed a slight increase close to the significance (Supplementary Fig. 3 c,d and f). These results confirm the role for BVR-A in the observed alterations found on insulin signaling and mitochondrial metabolism in GK rats. We also acknowledge few differences between the two models likely since the complete loss of BVR-A in BVR-A^−/−^ mice might accelerate and worsen the development of brain insulin resistance (increased IRS1 inhibition) and of mitochondrial impairment, resulting in the accumulation of oxidatively damaged proteins (PC and 3-NT). To further explore whether loss of BVR-A also has a role in the alterations of intracellular signalling pathways regulating cell energy metabolism and neuronal plasticity mechanisms found altered in GK rats, hippocampal samples were tested by label-free quantitative proteomics. We identified 989 proteins and isoforms with significant differences in expression (Supplementary Table 2). The Volcano plot highlights that proteins of the UPRmt canonical axis are among the most significantly upregulated in BVR-A^−/−^ mice (Hsp60, Hsp90, Sod2, Grp75, Lonp1) while proteins associated with cell energy metabolism (ATPase inhibitor F1, ATP synthase, Pip5k1a, Pkm, Ldhb and Mdh2) were downregulated (Fig. 3i). Remarkably, the IPA analysis displays similar altered pathways as those observed in GK rats (Fig. 3j). In addition, the comparison between the GK vs Wistar rats and BVR-A^−/−^ vs WT mice datasets reveals the overlapped alteration of 375 proteins, which account for the 27.7 % of the total amount of identified proteins (Fig. 3k). The comparative analysis between the Canonical Pathways identified by IPA in both mice and rats, supports the primary common dysfunction of mitochondrial metabolism (e.g., oxidative phosphorylation, sirtuin signaling pathway and TCA cycle), proteostasis network (chaperone-mediated autophagy signaling pathway, sirtuins signaling pathway and Nrf-2-mediated antioxidant response) and neuronal plasticity mechanisms (synaptogenesis, SNARE signaling, Rho/Rac-mediated pathways, LTP and actin dynamics) (Fig. 3l). Furthermore, the analysis of the Downstream Effects related to observed altered pathways reports the common alteration of several processes associated with neuronal development, growth, morphogenesis and plasticity (Fig. 3m). Accordingly, BVR-A^−/−^ mice exhibited impaired behavioral functions (Supplementary Fig. 3j-m).

### 3.4 Reduced BVR-A protein levels impairs mitochondrial bioenergetics in response to insulin

**Fig. 4:**
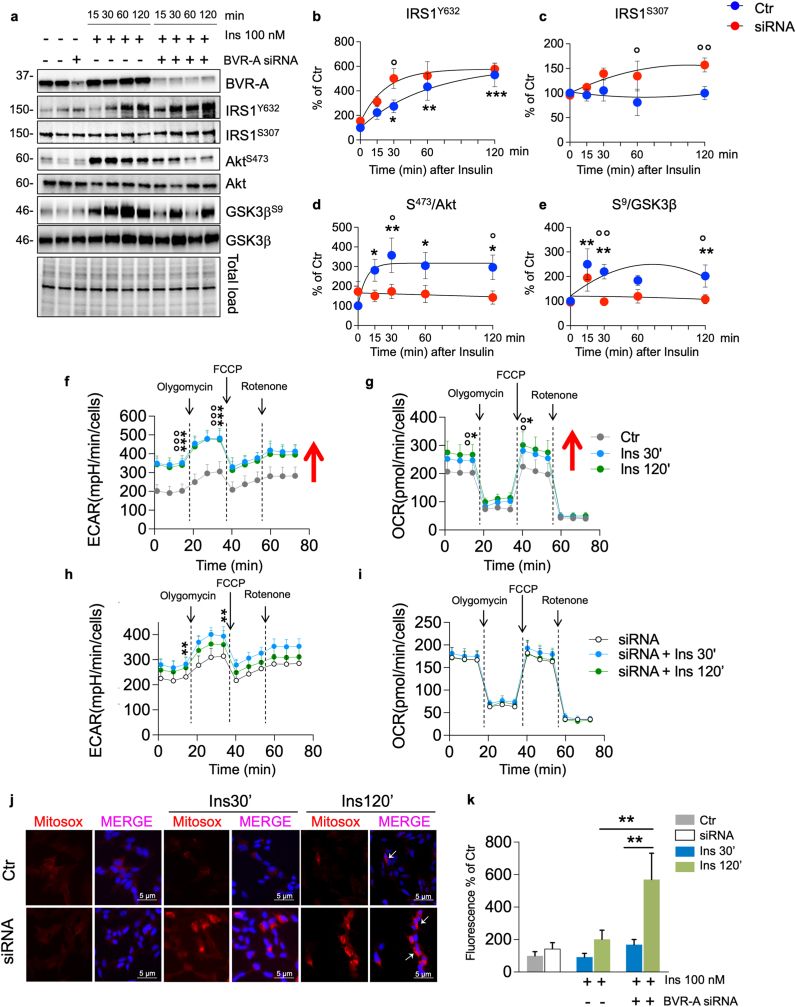
Reduced BVR-A protein levels impairs mitochondrial bioenergetic in response to insulin. Changes of BVR-A and of the insulin signaling pathway's proteins levels, i.e., IRS1, Akt and GSK3β were evaluated in Ctr and siRNA-treated cells stimulated with 100 nM insulin at different time points 15’, 30’, 60’ and 120’ minutes (n = 5–6 independent cultures/group). **(a)** Representative Western blot images and densitometric evaluation of **(b)** IRS1 activation evaluated as IRS1^Y632^ (*p = 0.03, Ctr vs 15’ Ins; *p = 0.0012, Ctr vs 30’ Ins; *p = 0.000016, Ctr vs 120’ Ins; °p = 0.01, 30’Ins vs 30’ siRNA+Ins) **(c)** IRS1 inhibition evaluated as IRS1^S307^ (°p = 0.03, 60’ Ins vs 60'siRNA+Ins; °p = 0.0078, 120’ Ins vs 120'siRNA+Ins) **(d)** Akt activation (evaluated as S^473^/Akt ratio; *p = 0.02, Ctr vs 15’ Ins; *p = 0.003, Ctr vs 30’ Ins; *p = 0.01, Ctr vs 60’ Ins; *p = 0.01, Ctr vs 120’ Ins; °p = 0.01, 30’ Ins vs 30'siRNA+Ins; °p = 0.03, 120’ Ins vs 120'siRNA+Ins) **(e)** GSK3β inhibition (evaluated as S^9^/GSK3β ratio; *p = 0.007, Ctr vs 15’ Ins; *p = 0.003, Ctr vs 30’ Ins; *p = 0.01, Ctr vs 120’ Ins; °p = 0.001, 30’ Ins vs 30’ siRNA+Ins; °p = 0.001, 120’ Ins vs 120'siRNA+Ins). Values are given as percentage of Ctr 0’ set as 100 %. Data are presented as means ± SEM (One-way ANOVA with Fisher's LSD test). **(f**–**i)** Bioenergetic profile evaluated by seahorse in Ctr and siRNA-treated SHSY-5Y cells stimulated with 100 nM Insulin for 30’ and 120’ minutes. **(f)** ECAR measured in Ctr cells (Ctr Baseline vs 30’Ins Baseline ***p = 1.76E-06; Ctr Baseline vs 120’Ins Baseline °°°p = 1.82E-05; Ctr Stressed vs 30’Ins Stressed ***p = 2.03E-05; Ctr Stressed vs 120’Ins Stressed °°°p = 6.18E-06) **(g)** OCR measured in Ctr cells (Ctr Baseline vs 30’Ins Baseline *p = 0.022; Ctr Baseline vs 120’Ins Baseline °°p = 0.0040; Ctr Stressed vs 30’Ins Stressed *p = 0,018; Ctr Stressed vs 120’Ins Stressed °°p = 0.0051). **(h)** ECAR measured in siRNA-treated cells (siRNA Baseline vs siRNA 30’Ins Baseline **p = 0.0057; siRNA Baseline vs siRNA 120’Ins Baseline p = 0.087; siRNA Stressed vs siRNA 30’Ins Stressed **p = 0.00057; siRNA Stressed vs siRNA 120’Ins Stressed p = 0.075). **(i)** OCR measured in siRNA-treated cells (siRNA Baseline vs siRNA 30’Ins Baseline OCR p = 0.34; siRNA Baseline vs siRNA 120’Ins Baseline p = 0.97; siRNA Stressed vs siRNA 30’Ins Stressed p = 0.34; siRNA Stressed vs siRNA 120’Ins Stressed p = 0.84). Arrows indicate the addition of inhibitors: Olygomycin, FCCP and Rotenone. Values are given as means ± SEM of 2 independent experiment with 8 replicates each (one-way ANOVA followed by Holm-Sidak post-hoc comparison test). Mitochondrial superoxide production was detected with MitoSOX. **(j)** Representative images of MitoSOX in Ctr and siRNA-treated cells stimulated with 100 nM for 30’ and 120’. MitoSOX in red and DAPI for nuclei visualization in blue. **(k)**. Immunofluorescence quantification of MitoSOX red was measured by ImageJ software (p = 0.0039, 120’ Ins vs 120'siRNA+Ins, p = 0.001, 30'siRNA+Ins vs 120’ siRNA+Ins). Values are given as percentage of Ctr 0’ set as 100 %. Data are presented as means ± SEM (one-way ANOVA with Fisher's LSD test). (For interpretation of the references to color in this figure legend, the reader is referred to the Web version of this article.)

To gain insights into the molecular mechanisms through which loss of BVR-A impairs insulin signaling activation and mitochondrial bioenergetics, we next utilized SHSY-5Y neuronal cells to study insulin signaling. First, the effect mediated by 100 nM insulin administration on the insulin intracellular signaling was tested in control cells and cells lacking BVR-A (siRNA-treated). We found that 100 nM insulin does not alter BVR-A protein levels (Fig. 4a). Rather, insulin caused the activation of IRS1 in control cells as highlighted by changes of IRS1^Y632^ (activation) with no changes in IRS1^S307^ (inhibition) (Fig. 4b and c). Loss of BVR-A promoted the hyper-activation of IRS1 (IRS1Y^632^) after 30 min, while a significant inhibition of IRS1 (IRS1^S307^) after 60 and 120 min was observed in siRNA-treated vs control cells (Fig. 4b). These data confirm for the first time, a role for BVR-A in regulating IRS1 activation [9], and that loss of BVR-A leads to the aberrant activation of IRS1 in response to insulin. If this phenomenon persists with time, the inhibition of IRS1 and the development of insulin resistance occurs (Fig. 4c). Downstream from IRS1, a rapid response to insulin stimulation was observed in the control cells, as demonstrated by increased Akt activation (S^473^/Akt) and GSK3β inhibition (S^9^/GSK3β) (Fig. 4 d,e). Strikingly, no changes were observed in cells lacking BVR-A (Fig. 4 d,e) – even in presence of hyper-active IRS1 (after 30 min) – suggesting that BVR-A is a key player regulating insulin signaling activation. Furthermore, the evaluation of mitochondrial complexes levels shows no significant changes (Supplementary Figs. 4b–f). Based on these results, we selected 30 and 120 min as reference time points for additional experiments aimed at evaluating mitochondrial bioenergetics in response to insulin. The rationale behind this choice is that: (1) the 30-min time point recapitulates an early phase condition, wherein reduced BVR-A protein levels parallel IRS1 hyper-activation and impaired Akt/GSK3β axis as observed in GK rats; while (2) the 120-min time point mirrors a later phase, manifesting the development of insulin resistance characterized by reduced BVR-A protein levels, IRS1 inhibition, and the persistent impairment of Akt/GSK3β axis as observed in BVR-A^−/−^ mice. To analyze the effect of silencing BVR-A on cellular metabolism, we assessed the energetic profile through Seahorse. As shown in Fig. 4f and g, insulin treatment leads to a significant increase of both ECAR and OCR (reporting glycolytic and respiration rate, respectively). In agreement with our observations, cells transfected with BVR-A siRNA weaken the insulin response with regard to ECAR while insulin effect is abolished on the OCR (Fig. 4h and i). In addition, SHSY-5Y cells were stained with MitoSOX™ Red to assess the levels of mitochondrial ROS (mtROS) to evaluate mitochondrial oxidative stress (Fig. 4j). 100 nM insulin promoted an increase of mtROS production after 120 min in siRNA-treated cells, while no differences were observed after 30 min (Fig. 4k). Having established that the loss of BVR-A significantly impacts insulin signaling activation and mitochondrial bioenergetics, we sought to identify potential factors responsible for the observed reduction in BVR-A levels within the hippocampus of GK rats. BVR-A protein levels negatively associates with HOMA-IR, and thus insulin resistance, in rats used in the current study. Previous studies by our group demonstrated reduced BVR-A levels in peripheral blood mononuclear cells (PBMC) of obese individuals [34]. Furthermore, we found a robust association between either higher circulating insulin levels or diminished cellular insulin sensitivity, with lower BVR-A levels [34]. To delve deeper into this relationship, we subjected SHSY-5Y cells to increased doses of insulin for 30 min, revealing that elevated insulin levels lead to a reduction in BVR-A protein levels (Supplementary Fig. 5b). Remarkably, an elevation of Grp75 was observed at the same doses (Supplementary Fig. 5c).

Overall, these results strengthen the hypothesis that reduced BVR-A protein levels impairs insulin signaling and mitochondrial bioenergetics leading to UPRmt activation, before overt insulin resistance, consistent with findings in GK rats and BVR^−/−^ mice.

### 3.5 BVR-A is a shuttle for GSK3β into the mitochondria in response to insulin

**Fig. 5:**
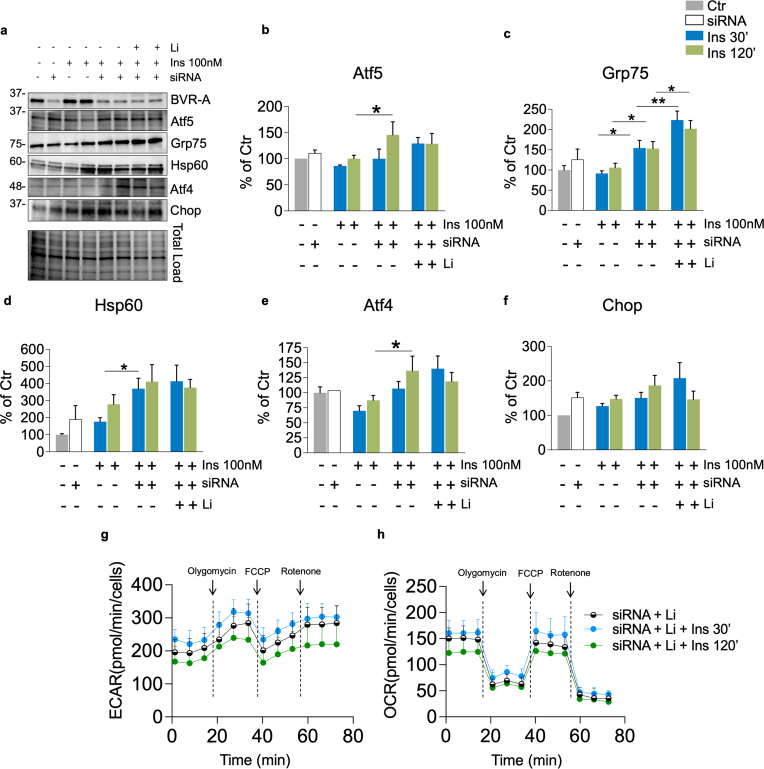
Reduced BVR-A protein levels trigger UPRmt activation in SHSY-5Y in response to insulin Changes of BVR-A and Unfolded Protein Response (UPRmt) proteins [Atf5, Grp75, Hsp60, Atf4, Chop] were evaluated in Ctr and siRNA-treated cells stimulated with 100 nM insulin for 30’ and 120’. Where indicated cells were pre-treated with 10 mM lithium chloride (LiCl) for 24 h before insulin administration (3–5 independent cultures/group) **(a)** Representative Western blot and densitometric evaluation of **(b)** Atf5 (p = 0.03, 120’Ins vs 120'siRNA+Ins) **(c)** Grp75 (p = 0.01, 30’Ins vs 30'siRNA+Ins; p = 0.04, 120’ Ins vs 120'siRNA+Ins; p = 0.0049, 30'siRNA+Ins vs 30'siRNA+Ins+ Li; p = 0.0040, 120'siRNA+Ins vs 120'siRNA+Ins+Li) **(d)** Hsp60 (p = 0.04, 30’ Ins vs 30'siRNA+Ins) **(e)** Atf4 (p = 0.03, 120’Ins vs 120'siRNA+Ins) **(f)** Chop. All densitometric values are given as percentage of Controls cells set as 100 %. Data are presented as means ± SEM (One-way ANOVA with Fischer LSD post-hoc test). **(g,h)** Bioenergetic profile evaluated by seahorse in siRNA-treated cells pretreated with 10 mM LiCl and stimulated with Insulin for 30’ and 120’ **(g)** ECAR and **(h)** OCR measured in siRNA-treated cells+Li. Arrows indicate the addition of inhibitors: Olygomycin, FCCP and Rotenone. Values are given as means ± SEM of 2 independent experiment with 8 replicates each (one-way ANOVA followed by Holm-Sidak post-hoc comparison test).

**Fig. 6:**
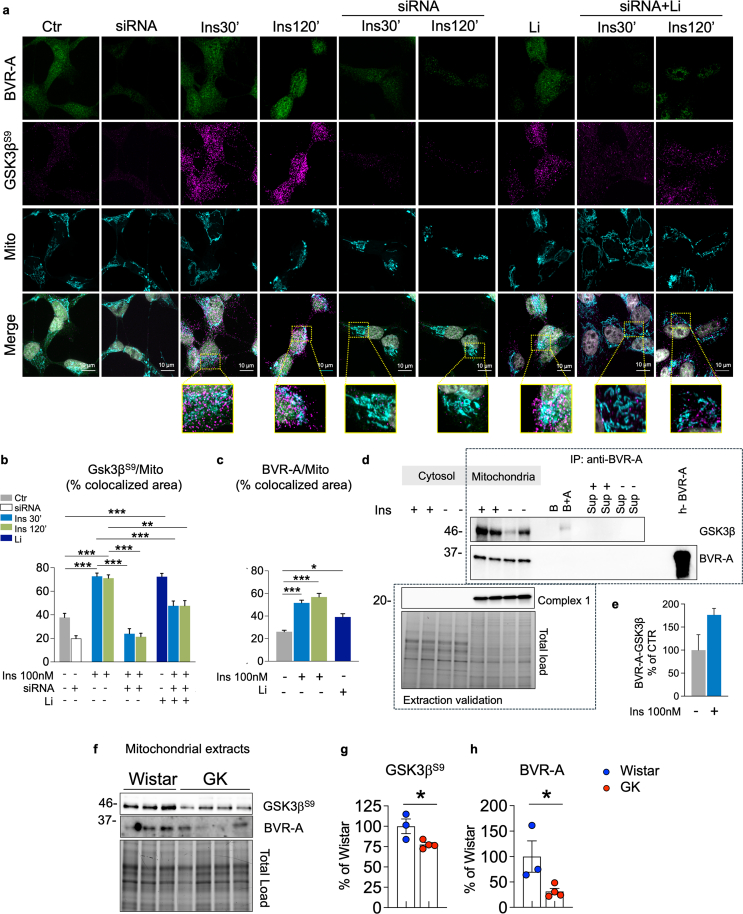
BVR-A is a shuttle for GSK3β into the mitochondria in response to insulin Changes of BVR-A and GSK3β were evaluated in Ctr and siRNA-treated cells stimulated with 100 nM insulin for 30’ and 120’. Where indicated cells were pre-treated with 10 mM lithium chloride (LiCl) for 24 h before insulin administration (3–5 independent cultures/group) (**a)** Representative confocal immunofluorescence images of BVR-A (green), Mitotracker (cyan) and GSK3β^S9^ (magenta) (scale bar: 10 μm, DAPI for nuclei visualization in grey) in Ctr and siRNA-treated cells stimulated with 100 nM insulin for 30’ and 120’. Where indicated cells were pre-treated with 10 mM lithium chloride (LiCl) for 24 h before insulin administration. **(b)** Quantification of GSK3β^S9^ and Mitotracker signals colocalization at single cell level in Ctr and siRNA-treated SHSY-5Y cells upon the treatments described above (p = 2E-07, Ctr vs 30’Ins; p = 8E-07, Ctr vs 120’Ins; p = 7E-12, 30’Ins vs 30'siRNA+Ins; p = 3E-12, 120’Ins vs 120'siRNA+Ins; p = 0.00007, 30’Ins vs 30'siRNA+Ins+Li; p = 0.0012, 120’Ins vs 120'siRNA+Ins+Li; p = 0.00001 Ctr vs Ctr+Li). For the analysis relative to siRNA-treated cells only silenced cells were took into consideration (**c)** Quantitative analysis showing the colocalization of BVR-A and Mitotracker signals at single cell level in Ctr cells upon the different treatments (p = 2E-06, Ctr vs 30’Ins; p = 8E-08, Ctr vs 120’Ins; p = 0.008 Ctr vs Li). Values are given as mean ± SEM (n = 6–11 cells from each condition from 3 independent experiments; One-way ANOVA with Sidak's multiple comparison test). **(d)** Representative Western blot images and **(e)** densitometric evaluation of the BVR-A/GSK3β complex isolated through the immunoprecipitation assay from SHSY-5Y mitochondrial extracts following stimulation with 100 nM insulin for 30’. Validation of the purity of the subcellular fractions was determined by examining Complex 1. To achieve a sufficient amount of mitochondrial proteins for the IP analyses extracts were pooled, resulting in a sample size of n = 2 for each condition. IP lanes description: lanes 1 and 2: SHSY-5Y stimulated with 100 nM insulin; lanes 3 and 4: CTR; lane 5: empty; lane 6: beads alone, lane 7: beads plus the anti-BVR-A primary antibody but no sample (B+A); lane 8: empty; lanes 9 to 12: Supernatant collected following beads magnetization; lane 13 and 14: empty; lane 15: Recombinant Human BVR-A protein used as positive control. Changes of BVR-A and GSK3β^S9^ levels evaluated in the hippocampal mitochondrial extract from Wistar (n = 3 independent samples) and GK (n = 4 independent samples) rats. **(f)** Representative Western blot images and densitometric evaluation of **(g)** GSK3β^S9^ (p = 0.03, GK vs Wistar) and **(h)** BVR-A (p = 0.04, GK vs Wistar). Values are given as percentage of Wistar set as 100 %. Data are presented as means ± SEM (Student *t*-test). Statistical significance was determined using Student t-test analysis (*p < 0.05, **p < 0.01). (For interpretation of the references to color in this figure legend, the reader is referred to the Web version of this article.)

**Fig. 7:**
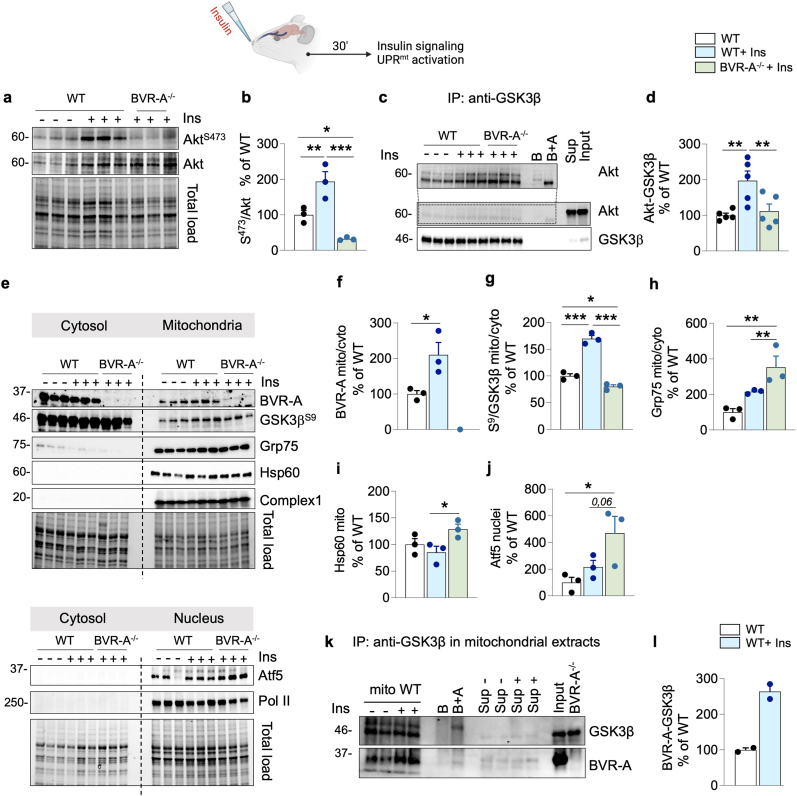
Loss of BVR-A impairs insulin signaling activation and GSK3β translocation into the mitochondria in the brain in response to insulin To confirm the activation of the insulin signaling in the brain Akt activation was evaluated in the hippocampus of WT and BVR-A^−/−^ mice treated with intranasal insulin for 30’ (n = 3 independent samples/group). **(a)** Representative Western blot of Akt^S473^, Akt and densitometric evaluation of **(b)** Akt activation (evaluated as S^473^/Akt ratio; p = 0.0096, WT vs WT+Ins; p = 0.0007, WT+Ins vs BVR-A−/− + Ins; p = 0.03, WT vs BVR-A^−/−^) **(c)** Representative Western blot images and **(d)** densitometric evaluation of the Akt/GSK3β complex isolated through the immunoprecipitation assay from the hippocampus from WT and BVR-A^−/−^ mice treated with intranasal insulin for 30’ (n = 5 independent samples/group). Lanes description: lanes 1 to 3: WT; lanes 4 to 6: WT stimulated with Intranasal Insulin; lanes 7 to 9: BVR-A^−/−^ stimulated with Intranasal insulin; lane 10: empty; lane 11: beads alone, lane 12: beads plus the anti-GSK3β primary antibody but no sample (B+A); lane 13: empty; lane 14: Supernatant collected following beads magnetization; and lane 14: Input. (p = 0.0045, WT vs WT+Ins; p = 0.0098, WT+Ins vs BVR-A^−/−^ +Ins). Evaluation of BVR-A, GSK3β and proteins of UPRmt i.e., Grp75, Hsp60 and Atf5 following subcellular fractionation of hippocampal samples collected from WT and BVR-A^−/−^ mice treated with intranasal insulin for 30’ (n = 3 independent samples/group). **(e)** Representative Western blot images and densitometric evaluation of **(f)** BVR-A (p = 0.03, WT vs WT+Ins), **(g)** GSK3β inhibition (evaluated as S^9^/GSK3β, p = 0.000038 WT vs WT+Ins; p = 0.000008, WT+Ins vs BVR-A^−/−^ + Ins; p = 0.02, WT vs BVR-A^−/−^ + Ins), **(h)** Grp75 (p = 0.0098, WT vs BVR-A^−/−^ + Ins; p = 0.04, WT+ Ins vs BVR-A^−/−^), **(i)** Hsp60 (p = 0.02, WT+Ins vs BVR-A^−/−^ + Ins), **(j)** Atf5 (p = 0.01, WT vs BVR-A^−/−^ + Ins; p = 0.06, WT+Ins vs BVR-A^−/−^). Purity of the subcellular fractions was determined by examining Complex I in the mitochondrial fraction and Polimerase II in the nuclear fraction by Western blot analysis. The densitometric values are given as percentage of WT set as 100 %. Data are presented as means ± SEM. One-way ANOVA with Fisher's LSD test. **(k)** Representative Western blot images and **(l)** densitometric evaluation of the BVR-A/GSK3β complex isolated through the immunoprecipitation assay from hippocampal mitochondrial extracts from WT mice treated with intranasal insulin for 30’. To achieve a sufficient amount of mitochondrial proteins for the IP analyses, hippocampal-derived extracts were pooled, resulting in a sample size of n = 2 for each condition. Lanes description: lanes 1 and 2: WT; lanes 3 and 4: WT stimulated with Intranasal Insulin; lane 5: empty; lane 6: beads alone, lane 7: beads plus the anti-GSK3β primary antibody but no sample (B+A); lane 8: empty; lanes 9 to 12: Supernatant collected following beads magnetization; lane 13: Input and lane 14: BVR-A−/− mice.

To further support the hypothesis for which insulin signaling alterations exacerbates mitochondrial dysfunctions and impair mitochondrial bioenergetics because reduced GSK3β S^9^ phosphorylation [50], we pre-treated SHSY-5Y cells with 10 mM lithium chloride — a potent GSK3β inhibitor acting independently of Akt [53,54] — prior to insulin administration. Initially, we focused on evaluating UPRmt canonical axis, because this branch was found altered in BVR-A^−/−^ mice. 100 nM insulin led to a significant increase of the UPRmt-coordinating transcription factor Atf5 after 120 min in siRNA-treated cells (Fig. 5b), while its downstream targets, i.e., Grp75 and Hsp60, exhibited significant increases as early as 30 min, suggesting a rapid activation of UPRmt in cells lacking BVR-A and stimulated with insulin (Fig. 5c and d). Atf4 show similar changes as those observed for Atf5 (Fig. 5e), while no alterations for Chop protein levels (Fig. 5f) were observed. Intriguingly, lithium did not reverse UPRmt activation; instead, we observed an even greater augmentation in Grp75 protein levels (Fig. 5c), implying that mitochondrial damage persists following lithium pre-treatment. This was further corroborated by evidence that lithium treatment does not restore ECAR and OCR in siRNA-treated cells in response to 100 nM insulin neither at 30 nor at 120 min (Fig. 5g and h). Considering the results obtained following lithium pre-treatment, it appears evident that an additional mechanism is behind these observations. As inhibited GSK3β regulates mitochondrial activity [50], and BVR-A functions as cytoplasmic shuttle for proteins between intracellular compartments [19], we hypothesized that the loss of BVR-A might be responsible for preventing GSK3β translocation into the mitochondria in response to insulin. Confocal microscopy analyses (Fig. 6a) confirmed our hypothesis showing that pGSK3β^S9^ levels increase in response to insulin (Supplementary Fig. 6a), and a greater translocation into the mitochondria can be observed either after 30 or 120 min (Fig. 6b). These effects were absent in BVR-A siRNA-treated cells (Fig. 6b). Intriguingly, lithium pre-treatment resulted in an increase of GSK3β^S9^ phosphorylation and its translocation into the mitochondria in control cells, in agreement with the presence of BVR-A (Fig. 6b). Conversely, lithium pre-treatment failed to promote an increased pGSK3β^S9^ translocation in cells lacking BVR-A in response to insulin (Fig. 6b). Furthermore, we observed a consistent overlap of BVR-A and Mitotracker signal in control cells both treated with insulin or lithium (Fig. 6c). Similar evidence in mitochondrial extracts isolated from SHSY-5Y cells treated with insulin and lithium are also provided (Supplementary Figs. 6b and c). To further support this molecular mechanism, we IPed BVR-A and evaluated the formation of the BVR-A/GSK3β complex in mitochondrial extracts isolated from SHSY-5Y cells treated with insulin (Fig. 6d). Our results show that a consistent increase of the complex can be detected into the mitochondria in response to insulin (Fig. 6d and e). Overall, these results provide for the first-time evidences that BVR-A translocates into the mitochondria in response to insulin likely functioning as a shuttle for pGSK3β^S9^ to foster oxidative phosphorylation. In addition, we evaluated the levels of BVR-A and pGSK3β^S9^ in hippocampal mitochondrial extracts from rats, and our results clearly show reduced levels of both proteins in mitochondrial extracts from GK rats, meaning that this is a conceivable mechanism occurring *in vivo* (Fig. 6f–h). To implement these findings, we examined the response to intranasal insulin administration (1 UI per nostril) in the hippocampus of WT and BVR-A^−/−^ mice. Insulin promoted the activation of insulin signaling in the hippocampus of WT but not in BVR-A^−/−^ mice, as demonstrated by the increased phosphorylation of Akt (Fig. 7b). Furthermore, the formation of the Akt-GSK3β complex is consistently increased in WT mice, while it is precluded in BVR-A^−/−^ mice (Fig. 7d). We then evaluated the cytosol-mitochondrial-nuclear trafficking of BVR-A, GSK3β and proteins of the canonical axis of UPRmt in response to insulin. We found that intranasal insulin led to increased BVR-A translocation within the mitochondria, aligning with the observed rise in pGSK3β^S9^ levels in WT mice (Fig. 7 e-g). GSK3β^S9^ translocation into the mitochondria was completely prevented in BVR-A^−/−^ mice, consistent with our previous results in cells and GK rats. Notably, the lack of pGSK3β^S9^ translocation in the mitochondria led to the activation of UPRmt in the hippocampus of BVR-A^−/−^ mice as demonstrated by the significant nuclear translocation of Atf5 along with the increased expression of Grp75 and Hsp60 into the mitochondria in response to insulin (Fig. 7 h-j). Finally, we evaluated the physical interaction between BVR-A and GSK3β within the mitochondria and our results show that increased BVR-A/GSK3β complex levels can be detected following intranasal insulin administration (Fig. 7k-l). These results provide compelling evidence supporting a link between insulin signaling and mitochondrial fitness through the interaction between BVR-A and pGSK3β^S9^, whereby alterations in this mechanism mediated by reduced BVR-A protein levels appear to be detrimental.

### 3.6 UPRmt activation in T2D and AD

**Fig. 8:**
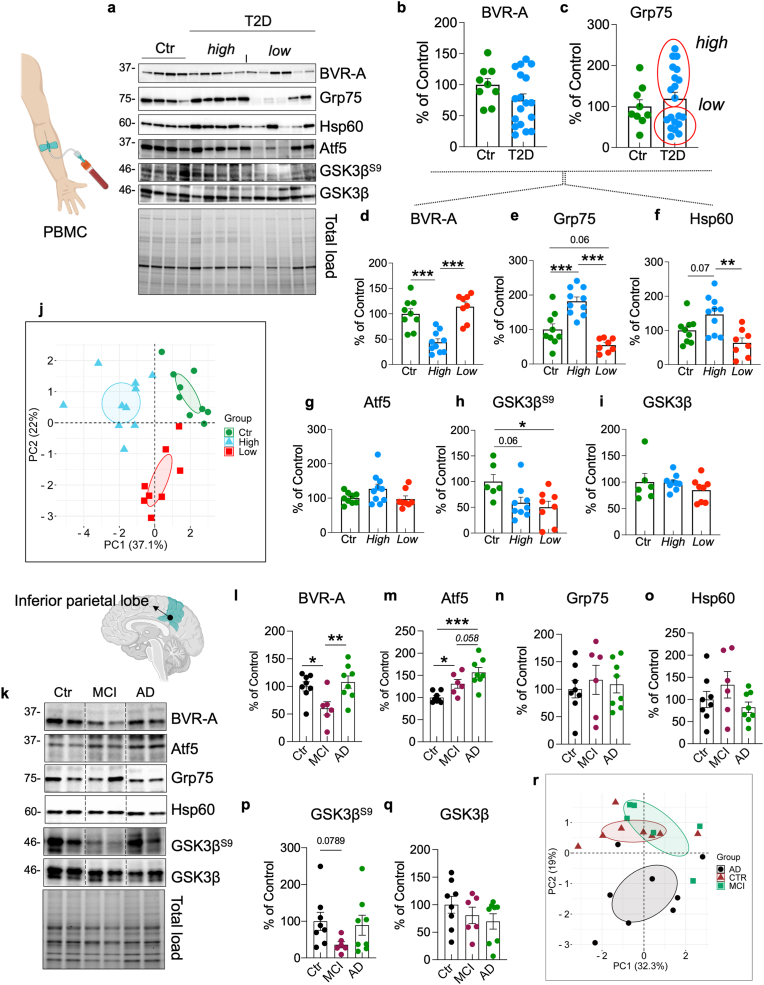
UPRmt activation in T2D and AD Changes of BVR-A, GSK3β inhibition and of the mitochondrial unfolded protein response (UPRmt) markers, i.e., Atf5, Grp75, Hsp60 evaluated in PBMC isolated from healthy (Ctr = 9) and T2D donors (n = 18). **(a)** Representative Western blot images of BVR-A, Grp75, Hsp60, Atf5, GSK3β and GSK3β^S9^ and densitometric evaluation of **(b)** BVR-A and **(c)** Grp75 **(d**–**j)** Densitometric values of BVR-A, Grp75, Hsp60, Atf5, GSK3β and GSK3β^S9^ are reported following categorizing our samples based on *high* and *low* Grp75 levels in T2D subjects. **(d)** BVR-A (p = 0.0002, Ctr vs High; p = 0,000019, High vs Low); **(e)** Grp75 (p = 0.0003, Ctr vs High; p = 0.0000011, High vs Low); **(f)** Hsp60 (p = 0.08, Ctr vs High; p = 0.0020 High vs Low); **(g)** Atf5; **(h)** GSK3β^s9^ (p = 0.06, Ctr vs High; p = 0.02 Ctr vs Low); **(i)** GSK3β. Data are presented as means ± SEM. Statistical analysis has been performed on each experiment by using one-way ANOVA followed by Tukey's multiple comparisons test. **(j)** Principal component analysis (PCA) performed on the results collected in human samples from Ctr and T2D donors. Changes of BVR-A, GSK3β inhibition and of the mitochondrial unfolded protein response (UPRmt) markers, i.e., Atf5, Grp75, Hsp60 evaluated in the inferior parietal lobule (IPL) of Control (Ctr) (n = 8), amnestic mild cognitive impairment (MCI, n = 6) and Alzheimer's disease (AD, n = 8) subjects. **(k)** Representative western blot images and densitometric evaluation of **(l)** BVR-A protein levels (p = 0.01, Ctr vs MCI; p = 0.005, MCI vs AD); **(m)** Atf5 (p = 0.02, Ctr vs MCI; p = 0.0001, Ctr vs AD; p = 0.058 MCI vs AD); **(n)** Grp75; **(o)** Hsp60; **(p)** GSK3β^S9^ (p = 0.07, Ctr vs MCI) and **(q)** GSK3β. Data are presented as means ± SEM (One-way ANOVA with Fisher's LSD test). **(r)** Principal component analysis (PCA) performed on the results collected in post-mortem brain samples from Ctr, amnestic MCI and AD subjects.

To examine the relevance of this mechanism in humans, we evaluated the activation of UPRmt in peripheral blood mononuclear cells (PBMCs) isolated from healthy (Ctr) and T2D donors, focusing on the disposition of BVR-A and Grp75. In our initial analysis, we did not observe any statistically significant differences between Ctr and T2D groups (Fig. 8b and c). However, upon closer examination of Grp75 levels, a noteworthy pattern emerged, revealing the presence of two distinct subgroups. One subgroup exhibited higher Grp75 levels, while the other subgroup is characterized by lower Grp75 levels (Fig. 8c). Hence, we categorized our samples based on *high* and *low* Grp75 levels to gain additional insights from these two distinct subgroups. We found that individuals with higher Grp75 levels show reduced BVR-A levels compared to Ctr subjects, along with a nearly significant increase of Hsp60 but no changes of Atf5 in PBMCs (Fig. 8 d-g). Furthermore, pGSK3β^S9^ levels were significantly reduced in both subgroups, despite no changes observed for total GSK3β protein levels (Fig. 8h and i). The multivariable regression model showed that, among patients with T2D, *high* levels of UPRmt proteins were associated with the concurrent use of multiple antidiabetic medications compared to those with *low* UPRmt protein levels [median (range) antidiabetic agents ongoing: *high* UPRmt: 2.5 (1–3) vs *low* UPRmt: 1 (1–2), p = 0.04]. This association was independent of gender, age, HbA1c values, BMI, and duration of diabetes (Table 5). This finding suggests that, to achieve similar glycemic control, these patients require targeting of multiple pathophysiological pathways. It could imply a more severe diabetic condition leading to greater cellular stress despite comparable disease duration. PCA analysis reveals distinct clusters for each of these 3 groups (Fig. 8j). Considering that T2D is a major risk factor for the development of AD, and that dysfunctional glucose metabolism, mitochondrial defects and insulin resistance are also key pathological hallmarks of AD brain, we performed the same analyses in post-mortem brain samples from MCI, AD and age-matched controls, to unravel whether AD development parallel alterations in UPRmt. We found that BVR-A protein levels are significantly reduced in MCI with respect to Ctr, while BVR-A levels are comparable to those of Ctr in AD subjects (Fig. 8l). However, an increase of 3-nitrotyrosine (3-NT) levels on BVR-A in both MCI and AD post-mortem brain was observed (Supplementary Fig. 7b), suggesting a consistent impairment despite unaltered protein levels in AD and in agreement with our previous work [55]. Alterations of BVR-A are associated with a significant increase of Atf5 both in MCI and AD brain, while no changes were observed for Grp75 and Hsp60 (Fig. 8m-o). Regarding GSK3β we observed a nearly significant decrease of pGSK3β^S9^ in MCI brain with respect to age-matched controls (Fig. 8p). Considering the average age of the subjects (∼90 yrs), these results describe a very late phase in the progression of the pathology supporting the failure of UPRmt activation in MCI and AD. PCA analysis performed by taking into consideration BVR-A, GSK3β, UPRmt markers, age, sex and MMSE scores, shows that only AD group can be identified by a distinct pattern (Fig. 8r). The explanation could be that, given an equal dysfunction in the activation of the UPRmt, most likely attributable to the advanced age of the study subjects, AD neuropathology becomes a distinguishing factor.

**Table 5:** Multivariate regression analysis. The number of anti-diabetic medications ongoing is the dependent variable.

	Unstandardized		Standardized	
	ß	Standard Deviation Error	ß	p-value
(Costant)	0.056	2.641		0.984
**High/Low UPRmt**	**1.199**	**0.410**	**0.658**	**0.017**
HbA1c	0.119	0.199	0.137	0.563
Gender	−0.752	0.468	−0.413	0.143
Age	−0.03	0.022	−0.346	0.192
Diabetes duration	0.043	0.024	0.481	0.113
BMI	0.025	0.048	0.123	0.698

R	R^2^	Corrected R^2^	Standard Deviation Error
0.794	0.631	0.385	0.714

## 4 Discussion

**Fig. 9:**
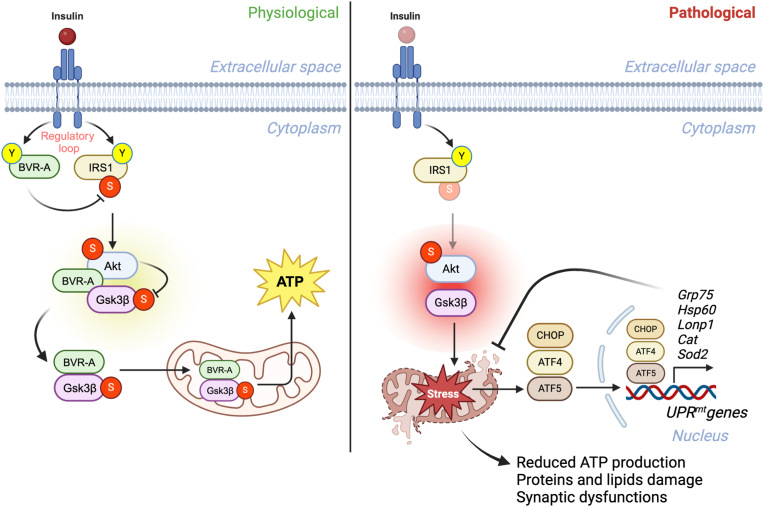
**Schematic representation of the proposed molecular mechanisms through which BVR-A links insulin signaling activation with mitochondrial metabolism.** Under Physiological Conditions (Left Panel): insulin binding to the insulin receptor (IR) initiates the phosphorylation of the insulin receptor substrate (IRS1) and BVR-A at Y residues. Phosphorylated BVR-A acts as a serine/threonine/tyrosine (S/T/Y) kinase, targeting inhibitory sites (S) on IRS1 to prevent IRS1 hyper-activation in response to insulin. Downstream from IRS1, Akt activation takes place. At this level, BVR-A serves as a scaffold protein, facilitating the physical interaction between Akt and GSK3β, leading to GSK3β phosphorylation at S9. Then, the BVR-A/pGSK3βS9 complex translocates into the mitochondria, promoting oxidative phosphorylation (OXPHOS) and ATP production to meet cellular metabolic demands. Under pathological conditions (right panel): reduced insulin sensitivity, leads to reduced BVR-A protein levels with the aim to increase IRS1 activation (by reducing the BVR-A-mediated inhibitory effect). However, this event creates a dual challenge downstream from IRS1. Loss of BVR-A compromises Akt-mediated inhibition of GSK3β, hindering the translocation of pGSK3βS9 into the mitochondria. This disruption results in mitochondrial stress and reduced ATP production. The ensuing cellular stress activates the mitochondrial unfolded protein response (UPRmt), prompting the translocation of transcription factors such as ATF5, ATF4, and CHOP into the nucleus. These factors promote the transcription of genes involved in the cellular stress response, including Grp75, HSP60, Lonp1, Cat, and Sod2, among others. Upregulation of these proteins aims to restore mitochondrial function, enhance ATP production, reduce oxidative stress-induced damage to proteins and lipids, and support synaptic plasticity mechanisms. Arrows: activation; lines:inhibition. The scheme has been created with BioRender.com.

The current study unveils the pivotal role played by BVR-A and GSK3β in the intricate regulation of brain energy metabolism in response to insulin, a process that undergoes significant alterations throughout the progression of T2D. This dysregulation poses a substantial risk factor for the onset and development of dementia and AD. Our research work presents a groundbreaking finding, illustrating that the reduction of BVR-A protein levels is sufficient to compromise the insulin-mediated response, reminiscent of a state akin to insulin resistance. Notably, this impairment manifests in the disruption of mitochondrial bioenergetics in the brain which precedes the accumulation of well-established markers of insulin resistance, such as inhibited IRS1. Furthermore, our study highlights the critical role of BVR-A in promoting GSK3β phosphorylation and its subsequent translocation into the mitochondria (Fig. 9).

Insulin signaling regulates mitochondrial DNA and protein synthesis and potently stimulates mitochondrial oxidative capacity and ATP production in peripheral tissues [56,57]. Proper insulin signaling activation in the brain has been associated with improved metabolism and better cognitive functions [58]. Several studies have also indicated that insulin treatment can attenuate diabetes-related brain-resident mitochondrial alterations, emphasizing the role of insulin in preserving mitochondrial functions [59,60]. Rather, brain insulin resistance development has been associated with a failure of mitochondria activities in a number of diseases, including AD [59].

Within this context, mitochondria capacity to adapt to cellular stressors, is a crucial aspect for optimal metabolism and to keep brain cells in good shape [61]. Damaged mitochondria induce several adaptive stress responses such as UPRmt and the integrated stress response (ISR), that initially aims to re-establish cellular homeostasis by preserving mitochondrial health and facilitating the fulfillment of energy requirements [62]. However, this protective role weakens with the progression of the pathology [62]. The UPRmt has been traditionally seen as a transcriptional response that increases the expression of mitochondrial chaperones in response to protein misfolding [63,64]. Recent lines of evidence have revealed that the UPRmt protects cells from a broader range of mitochondrial stresses, including OXPHOS dysfunction, perturbed protein import arising from mitochondrial protein misfolding, ATP depletion, or dissipation of mitochondrial inner membrane potential [65].

Our findings demonstrate that T2D development significantly impacts the brain, characterized by the impairment of OXPHOS complex activity, along with diminished levels of proteins integral to key metabolic pathways. In parallel, the activation of the UPRmt occurs, manifesting with the up regulation of mitochondrial complexes levels and proteins involved in the antioxidant responses. This observation, likely describing an early phase during the development of T2D-associated neuropathology, has not been described before.

Indeed, studies in literature suggest that UPRmt-response is weakened in T2D and is responsible for mitochondrial and cellular dysfunctions as well as increased oxidative stress [66]. In a model of high fat diet-induced brain insulin resistance, a disrupted mitochondrial stress response led to mitochondrial dysfunction, excessive autophagy, and increased weight gain [61]. Short-term intranasal insulin application restored the expression of Atf4, Chop, Hsp60, Hsp10, ClpP, and Lonp1, suggesting that insulin signaling regulates mitochondrial stress response and ensures proper mitochondrial function [61]. Additionally, T2D mice exhibit a reduction of the Hsp60 mRNA in the hypothalamus that is sufficient to induce hypothalamic insulin resistance [[67], [68], [69]]. Furthermore, Hsp60 mRNA are decreased in post-mortem brain samples from T2D patients [67]. Our data collected on MCI and AD post-mortem brain samples further support the weaking of UPRmt with the progression of the pathology [70], whereby persistently elevated Atf5 levels parallel no changes on the downstream targets and mostly important are associated with clinically relevant neuropathology. Nevertheless, it is also important to underly that the abnormal activation of UPRmt leads to cells undergoing continuous mitochondrial recovery [51]. This process may elevate the risk of accumulating misfolded proteins with aging, playing a direct role in age-related deterioration [51]. Therefore, the precise regulation of UPRmt becomes crucial [51]. Identifying these early alterations in the brain might be valuable for better characterizing the impact of T2D on the brain and for identifying subjects at a higher risk of developing neurodegenerative diseases.

Data collected in the current study delineate a novel molecular mechanism altered during the onset and development of T2D, elucidating the prominent connection between the failure of insulin signaling activation and the consequent mitochondrial dysfunction in the brain. This mechanism potentially establishes a link between peripheral and central alterations. First, T2D development promotes a reduction of BVR-A protein levels that alters the activation of the insulin signaling resulting in reduced Akt-mediated inhibition of GSK3β in the brain. Second, loss of BVR-A hinders the translocation of pGSK3β^S9^ into the mitochondria. Hence, BVR-A functions both as scaffold protein facilitating the physical interaction between Akt and GSK3β and as a shuttle for pGSK3β^S9^. These observations expand our understanding of BVR-A functions, showcasing its multifaceted role in insulin signaling through its primary and secondary structural features, as highlighted in previous studies [19,23]. Third, the reduced phosphorylation and inactivation of GSK3β coupled with its diminished mitochondrial translocation represents a detrimental event for brain cells, that disrupt the efficiency of the OXPHOS [71], and consequently impacts on energy production.

Previous studies show that reduced GSK3β phosphorylation impairs mitochondrial biogenesis, reduces mitochondrial dynamics, and increases mitochondrial permeability [71]. Remarkably, pGSK3β^S9^ plays a critical role in the maintenance of the activities of OXPHOS complexes thus fostering oxidative phosphorylation and ATP production, while reducing ROS generation [71]. Additionally, pGSK3β^S9^ prevents the opening of the mitochondrial permeability transition pore (mPTP) that promotes cell death [71]. Furthermore, phosphorylated GSK3β is prevented from interacting with the mitochondrial complex II, an event that would otherwise trigger increased ROS production [71]. Thus, the phosphorylation of GSK3β at Ser9 is a critical neuroprotective event.

Intriguingly, in the absence of BVR-A-mediated pGSK3β^S9^ translocation into the mitochondria - as seen in GK rats, BVR-A^−/−^ mice and in Li-treated cells - mitochondrial activity is consistently compromised and stress-induced responses, i.e., UPRmt, are activated. Experiments performed with LiCl clearly suggest that the phosphorylation of GSK3β, obtained in an insulin-independent manner, is not sufficient to prevent mitochondrial dysfunctions when BVR-A levels are reduced. These lines of evidence emphasize the novel intracellular mechanism linking insulin signaling and mitochondrial bioenergetics, that, instead, appears disrupted in the brain during T2D development.

Since BVR-A is the enzyme responsible for bilirubin production, this rise questions on whether a co-involvement of bilirubin in the observed brain alterations occurs. Bilirubin was recently reported to be a ligand for peroxisome proliferator-activated receptor alpha (PPARα) that induces gene responsiveness [72], which enhance mitochondrial activity, improves insulin resistance and obesity. Moreover, bilirubin possesses a strong antioxidant and anti-inflammatory effects that might improve glucose and insulin sensitivity [73]. Higher bilirubin levels were associated with enhanced insulin sensitivity, and glucose metabolism as well as decreased prevalence of T2D [73]. In addition, from a molecular point of view, an intriguing aspect relies with the fact that insulin favors IR-mediated BVR-A phosphorylation, that, other than promoting BVR-A kinase activity also increases reductase activity and bilirubin production [21]. This aspect further supports the key and pleiotropic role for BVR-A in linking insulin signaling with mitochondrial metabolism through multiple ways. Notwithstanding with that, understanding the role for bilirubin deserve further research and is far from the scope of our paper.

Rather, the significance of the findings described in our paper extends beyond the regulation of insulin signaling and mitochondrial metabolism, intersecting with the regulation of neuronal plasticity mechanisms. Mitochondria play a key role in supplying energy to fuel neuronal processes and their energy production is needed to support molecular mechanisms associated with synaptic transmission and plasticity [74]. Instead, mitochondrial bioenergetic failure leads to ageing-associated changes responsible for brain dysfunctions, cellular senescence, and neurodegeneration [[75], [76], [77], [78], [79], [80]]. Pioneering studies suggest that inhibition of GSK3β is critical in facilitating the induction of long-term potentiation (LTP), while increased activity promotes long-term depression (LTD) and controls metaplasticity in the hippocampal region [81]. Similarly, a recent work from Snyder-Paul group and ours highlighted that loss of BVR-A promotes learning and memory deficits and that BVR-A physically interacts with key molecules in focal adhesion signaling to mediate synaptic signaling in the hippocampus [29]. In agreement, insulin improves BVR-A and GSK3β S9 phosphorylation and promotes better cognitive and learning functions in animal models of aging and AD [9].

Lastly, we want to stress the fact that abnormal circulating insulin levels during the early phases of T2D development (pre-diabetic state) might be responsible for reduced BVR-A in the brain contributing to cognitive dysfunctions. Peripheral insulin resistance and thus elevated circulating insulin levels are a significant modulator of cognitive decline in persons at risk for T2D, even independently of glycemia, suggesting that not elevated blood glucose *per se* but rather insulin resistance is an important factor driving cognitive decline [5,82]. Indeed, extended periods of heightened peripheral insulin levels result in the downregulation of insulin transport into the brain, potentially influencing cognitive function [83]. In addition, peripheral insulin resistance has been associated with AD markers accumulation [82,[84], [85], [86]]. GK rats show (i) an impaired glucose metabolism under the IPGTT underlying a state of insulin resistance, (ii) a consistently impaired insulin signaling activation in the brain, characterized by IRS1 hyper-activation but reduced downstream effects, and (iii) impaired cognitive functions. Diminished levels of BVR-A observed in GK rats hippocampus strongly correlate with a well-accepted index of insulin resistance, i.e., HOMA-IR and likely serve as a compensatory mechanism to enhance insulin signaling activation in the setting of reduced insulin sensitivity [35]. As previously documented by our group, BVR-A levels exhibit dynamic changes during the OGTT in humans that depends on circulating insulin levels [35]. Specifically, BVR-A protein levels in PBMC initially decrease to facilitate the activation of IRS1, and then increase to support downstream intracellular signaling activation [35]. Variations in BVR-A protein levels during the OGTT are more pronounced in individuals with lower insulin sensitivity. Our hypothesis posits that, owing to decreased insulin sensitivity, cells attempt to enhance signaling activation through the modulation of BVR-A. However, this phenomenon is a double-edged sword, as persistently low BVR-A levels negatively impact insulin signaling activation downstream from IRS1, as evidenced in our study. In support of that, significantly lower BVR-A levels were observed following high insulin levels in SH-SY5Y cells as well as in obese [33] and T2D individuals [34].

## Conclusions

This nuanced exploration sheds light on novel insights into the intricate interplay between insulin and mitochondrial metabolism in the brain (Fig. 9), unraveling potential therapeutic targets for mitigating the cascading effects of brain insulin resistance development in the context of neurodegenerative diseases.

## CRediT authorship contribution statement

**Chiara Lanzillotta:** Writing – review & editing, Writing – original draft, Visualization, Investigation, Formal analysis, Data curation. **Antonella Tramutola:** Writing – review & editing, Investigation, Formal analysis. **Simona Lanzillotta:** Investigation. **Viviana Greco:** Writing – review & editing, Methodology, Investigation, Formal analysis. **Sara Pagnotta:** Investigation. **Caterina Sanchini:** Investigation. **Silvia Di Angelantonio:** Methodology, Investigation, Formal analysis. **Elena Forte:** Writing – review & editing, Resources, Methodology, Investigation. **Serena Rinaldo:** Investigation, Formal analysis. **Alessio Paone:** Investigation. **Francesca Cutruzzolà:** Resources, Methodology, Investigation. **Flavia Agata Cimini:** Resources, Methodology, Investigation. **Ilaria Barchetta:** Methodology, Investigation, Formal analysis. **Maria Gisella Cavallo:** Writing – review & editing, Resources, Methodology. **Andrea Urbani:** Resources, Methodology. **D. Allan Butterfield:** Writing – review & editing, Resources. **Fabio Di Domenico:** Writing – review & editing, Resources, Methodology, Investigation, Formal analysis. **Bindu D. Paul:** Writing – review & editing, Resources. **Marzia Perluigi:** Writing – review & editing, Resources. **Joao M.N. Duarte:** Writing – review & editing, Resources, Investigation, Formal analysis. **Eugenio Barone:** Writing – review & editing, Writing – original draft, Visualization, Supervision, Resources, Methodology, Investigation, Funding acquisition, Formal analysis, Data curation, Conceptualization.

## Declaration of competing interest

The authors declare that they have no known competing financial interests or personal relationships that could have appeared to influence the work reported in this paper.

## Data Availability

Data will be made available on request.
