# Supplementary material for: Biliverdin Reductase-A integrates insulin signaling with mitochondrial metabolism through phosphorylation of GSK3β
Source: Redox Biol. 2024 Jun 1;73:103221. doi: 10.1016/j.redox.2024.103221 (PMC11190564; doi:10.1016/j.redox.2024.103221)
Supplement: Multimedia component 1 [file mmc1.pdf]

Supplementary Materials for

BILIVERDIN REDUCTASE-A INTEGRATES INSULIN SIGNALING WITH MITOCHONDRIAL METABOLISM THROUGH PHOSPHORYLATION OF GSK3β

Chiara Lanzillotta[1], Antonella Tramutola[1], Simona Lanzillotta[1], Viviana Greco[2,3], Sara Pagnotta[1], Caterina Sanchini[4], Silvia Di Angelantonio[4,5], Elena Forte[1], Serena Rinaldo[1], Alessio Paone[1], Francesca Cutruzzolà[1], Flavia Cimini[6], Ilaria Barchetta[6], Maria Gisella Cavallo[6], Andrea Urbani[2,3], D Allan Butterfield[7], Fabio Di Domenico[1], Bindu D Paul[8,9,10,11], Marzia Perluigi[1], Joao MN Duarte[12,13] and Eugenio Barone[1,*]

[1]Department of Biochemical Sciences "A. Rossi-Fanelli", Sapienza University of Rome, Italy;
[2]Department of Basic Biotechnology, Perioperative and Intensive Clinics, Faculty of Medicine and Surgery, Catholic University of the Sacred Heart, L.go F.Vito 1, 00168 Rome, Italy;
[3]Fondazione Policlinico Universitario A. Gemelli IRCCS, L.go A.Gemelli 8, 00168 Rome, Italy;
[4]Center for Life Nano- & Neuro-Science, Istituto Italiano di Tecnologia, 00161 Rome, Italy;
[5]Department of Physiology and Pharmacology, Sapienza University of Rome, Italy;
[6]Department of Experimental Medicine, Sapienza University of Rome, Italy;
[7]Sanders-Brown Center on Aging, Department of Chemistry, University of Kentucky, Lexington, KY, USA;
[8]The Solomon H. Snyder Department of Neuroscience, Johns Hopkins University School of Medicine, Baltimore, MD, USA;
[9]Department of Pharmacology and Molecular Sciences, Johns Hopkins University School of Medicine, Baltimore, MD, USA;
[10]Department of Psychiatry and Behavioral Sciences, Johns Hopkins University School of Medicine, Baltimore, MD, USA;
[11]Lieber Institute for Brain Development, Baltimore, MD, USA;
[12]Department of Experimental Medical Science, Faculty of Medicine, Lund University, Sweden;
[13]Wallenberg Centre for Molecular Medicine, Lund University, Lund, Sweden;

Correspondence:

Prof. Eugenio Barone, Department of Biochemical Sciences "A. Rossi-Fanelli", Sapienza University of Rome, Italy. eugenio.barone@uniroma1.it

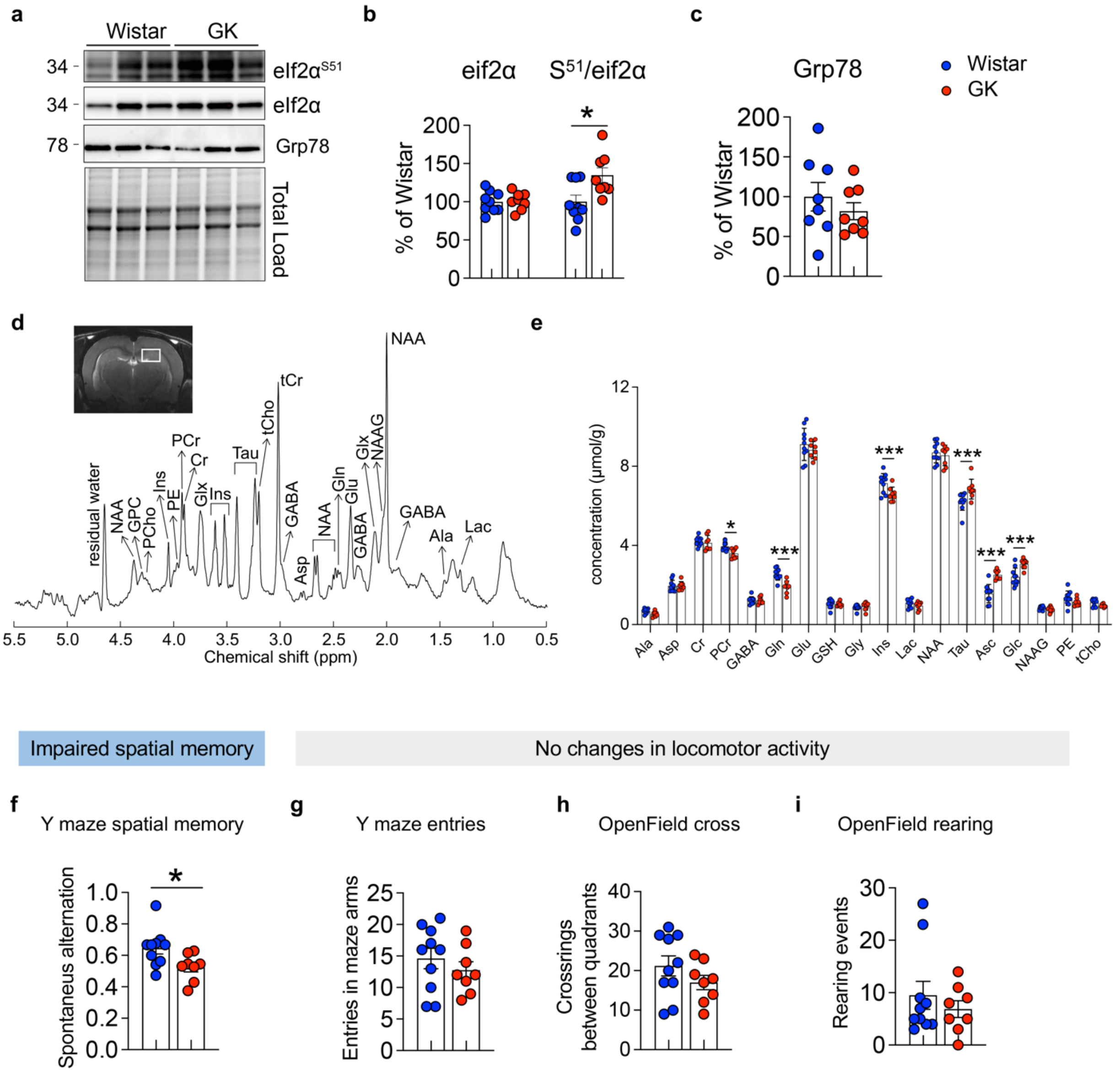

Supplementary Figure 1. GK rats are characterized by increased activation of eif2α and impairment of metabolic pathways that results in the alterations of cognitive function

Changes of eif2α and Grp78 were evaluated in the hippocampus of Wistar (n=8-9 independent samples) and GK (n= 7-8 independent samples) rats. **(a)** Representative Western blot images and densitometric evaluation of **(a)** eif2α protein levels (p=0.89, GK vs Wistar), and eif2α activation

(evaluated as p eif2α ^{S51}/ eif2α ratio; p=0.01, GK vs Wistar). The densitometric values are given as percentage of Wistar set as 100%. Data are presented as means ± SEM. Statistical significance was determined using Student t-test analysis (*p < 0.05, **p < 0.01). **(d)** Typical spectrum obtained from the rat hippocampus using SPECIAL at 14.1 T. For signal enhancement, a Gaussian apodization (gf = 0.08, gfs = 0.02) was applied prior Fourier transformation. The inset brain image displays the location of the volume of interest for MRS. **(e)** Metabolite concentrations. Legend: Lac, lactate; Ala, alanine; GABA, γ-aminobutyrate; NAA, *N*-acetylaspartate; NAAG, *N*-acetylaspartylglutamate; Glu, glutamate; Gln, glutamine; Glx, Glu+Gln; Asp, aspartate, Cr, creatine; PCr, phosphocreatine; tCr=Cr+PCr; tCho, total choline-containing compounds; Tau, taurine; Ins, *myo*-inositol; PE, phosphorylethanolamine; Pcho, phosphorylcholine; GPC, glycerophosphorylcholine. **(e,f)** Data of Y-maze test. **(e)** spontaneous alternation (p=0.02, GK vs Wistar) and **(f)** total entries in maze arms. **(g,h)** Data of exploration of the open-field (OF) arena. **(g)** crossrings between quadrants and **(h)** rearing events. Data presented as mean ± SEM. Statistical significance was determined using Student t-test (*p < 0.05).

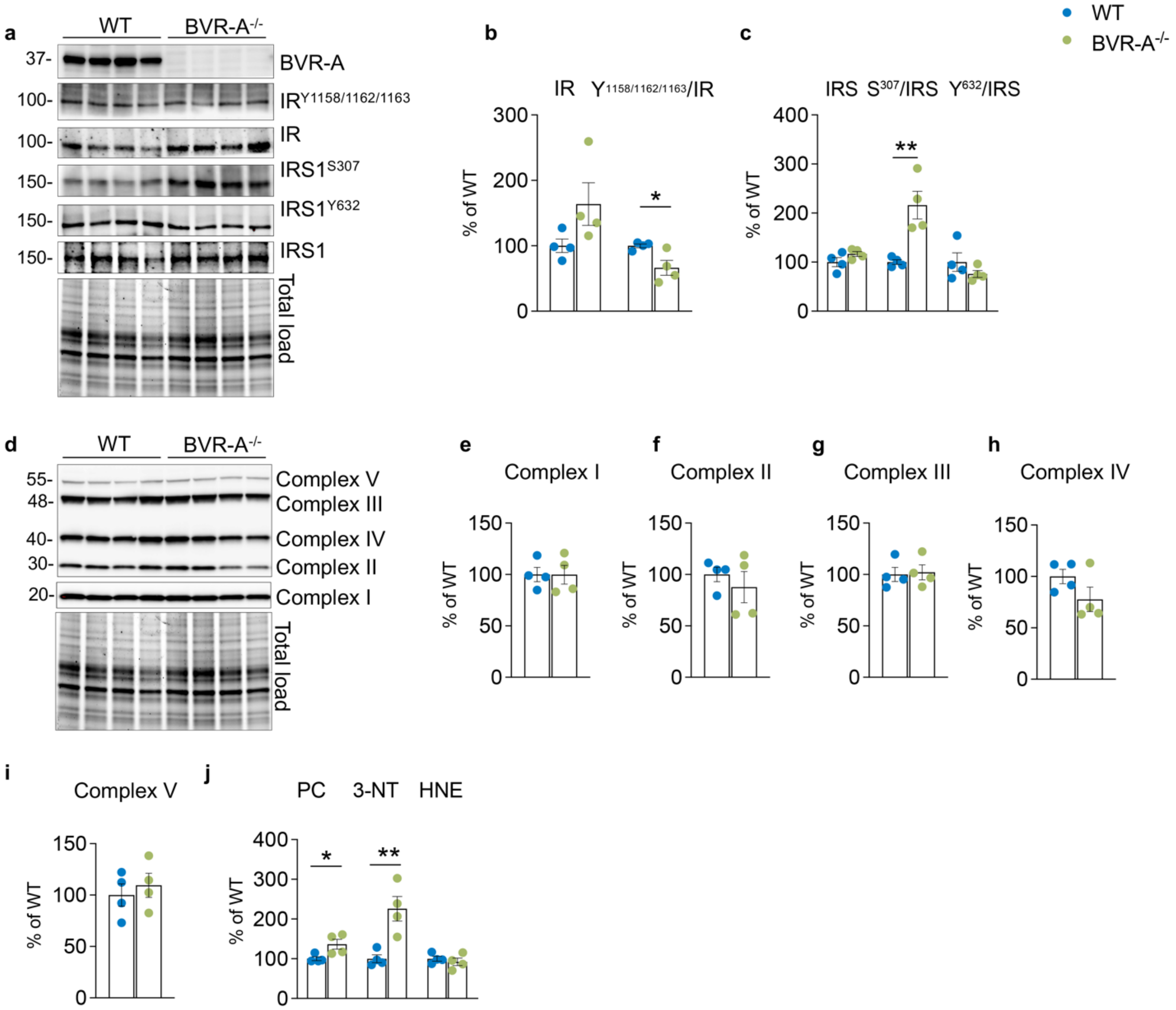

Supplementary Figure 2. BVR-A$^{-/-}$ mice show alterations of the insulin signaling pathway in the brain

Changes of the insulin signaling pathway's proteins levels, i.e., IR, IRS1, in the hippocampus of WT (n=4 independent samples) and BVR-A$^{-/-}$ (n=4 independent samples) mice. **(a)** Representative Western blot images and densitometric evaluation of **(b)** IR protein levels (p=0.11, WT vs BVR-A$^{-/-}$), and IR activation (evaluated as pIR$^{Y1158/1162/1163}$/IR ratio; p=0.01 WT vs BVR-A$^{-/-}$); **(c)** IRS1 protein levels (p=0.16, WT vs BVR-A$^{-/-}$), IRS1 inhibition (evaluated as S^{307}/IRS1 ratio; p=0.0067, WT vs BVR-A$^{-/-}$) and IRS1 activation (evaluated as Y^{632}/IRS1 ratio; p=0.27 WT vs BVR-A$^{-/-}$). **(d)** Representative Western blot images and densitometric evaluation of **(e)** Complex I (subunit

NDUFB8), **(f)** Complex II (subunit SDHB), **(g)** Complex III (subunit UQCRC2), **(h)** Complex IV (subunit MTCO1), and **(i)** Complex V (subunit ATP5A). **(j)** Densitometric evaluation of oxidative stress markers, i.e., protein carbonyls (PC) (p=0.03, WT vs BVR-A$^{-/-}$), 3-nitrotyrosine (3-NT) (p=0.0082, WT vs BVR-A-/-) and proteins-bound 4-hydroxyl-2-nonenals (HNE) (p=0.50, WT vs BVR-A$^{-/-}$) evaluated in the hippocampus of WT (n=4 independent samples) and BVR-A$^{-/-}$ (n=4 independent samples) mice. Values are given as percentage of WT set as 100%. Data are presented as means ± SEM. Statistical significance was determined using Student t-test (*$p < 0.05$).

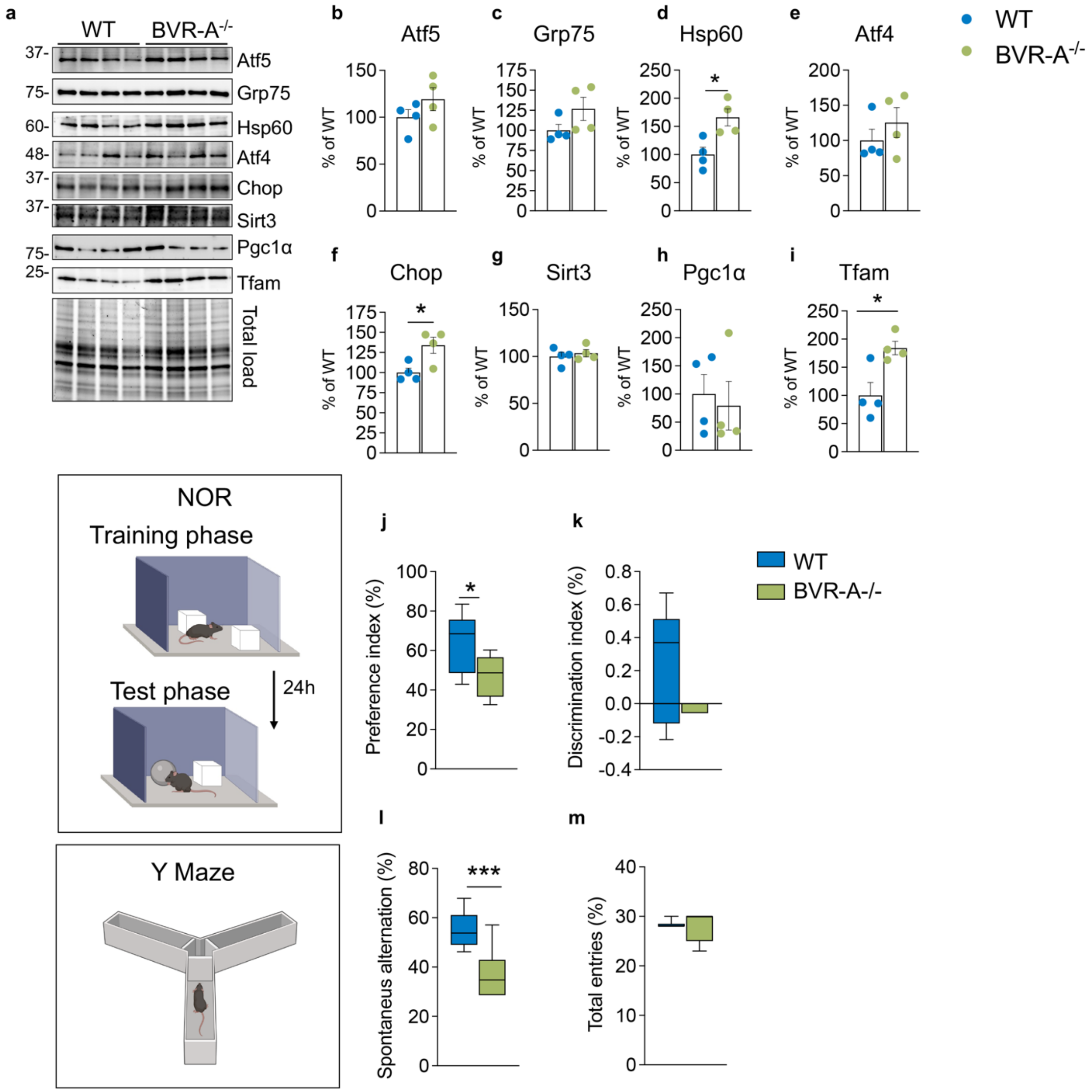

Supplementary Figure 3. Unfolded Protein Response and Cognitive Functions in BVR-A$^{-/-}$ mice

Changes of the Unfolded Protein Response (UPRmt) proteins [Atf5, Grp75, Hsp60, Atf4, Chop, Sirt3, Pgc1α and Tfam] were evaluated in the hippocampus of WT (n=4 independent samples) and BVR-A$^{-/-}$ (n=4 independent samples) mice. **(a)** Representative Western blot images and densitometric evaluation of **(b)** Atf5 (p=0.23, WT vs BVR-A$^{-/-}$), **(c)** Grp75 (p=0.14, WT vs BVR-A$^{-/-}$), **(d)** Hsp60 (p=0.01, WT vs BVR-A$^{-/-}$), **(e)** Atf4 (p=0.3, WT vs BVR-A$^{-/-}$), **(f)** Chop (p=0.02,

WT vs BVR-$A^{-/-}$), **(g)** Sirt3 (p=0.5, WT vs BVR-$A^{-/-}$), **(h)** Pgc1α (p=0.7, WT vs BVR-$A^{-/-}$) and **(i)** Tfam (p=0.01, WT vs BVR-$A^{-/-}$). Values are given as percentage of WT set as 100%. Data are presented as means ± SEM. Statistical significance was determined using Student t-test (*$p < 0.05$). Defects in cognitive and memory functions in BVR-$A^{-/-}$ mice. **(j,k)** Data of the novel object recognition test. **(j)** Preference index (p=0.02, WT vs BVR-$A^{-/-}$) and **(k)** Discrimination index. **(l,m)** Data of Y-maze test. **(l)** spontaneous alternation (p=0.0008, WT vs BVR-$A^{-/-}$) and **(m)** total entries. Data presented as mean ± SEM. Statistical significance was determined using Student t-test (*$p < 0.05$).

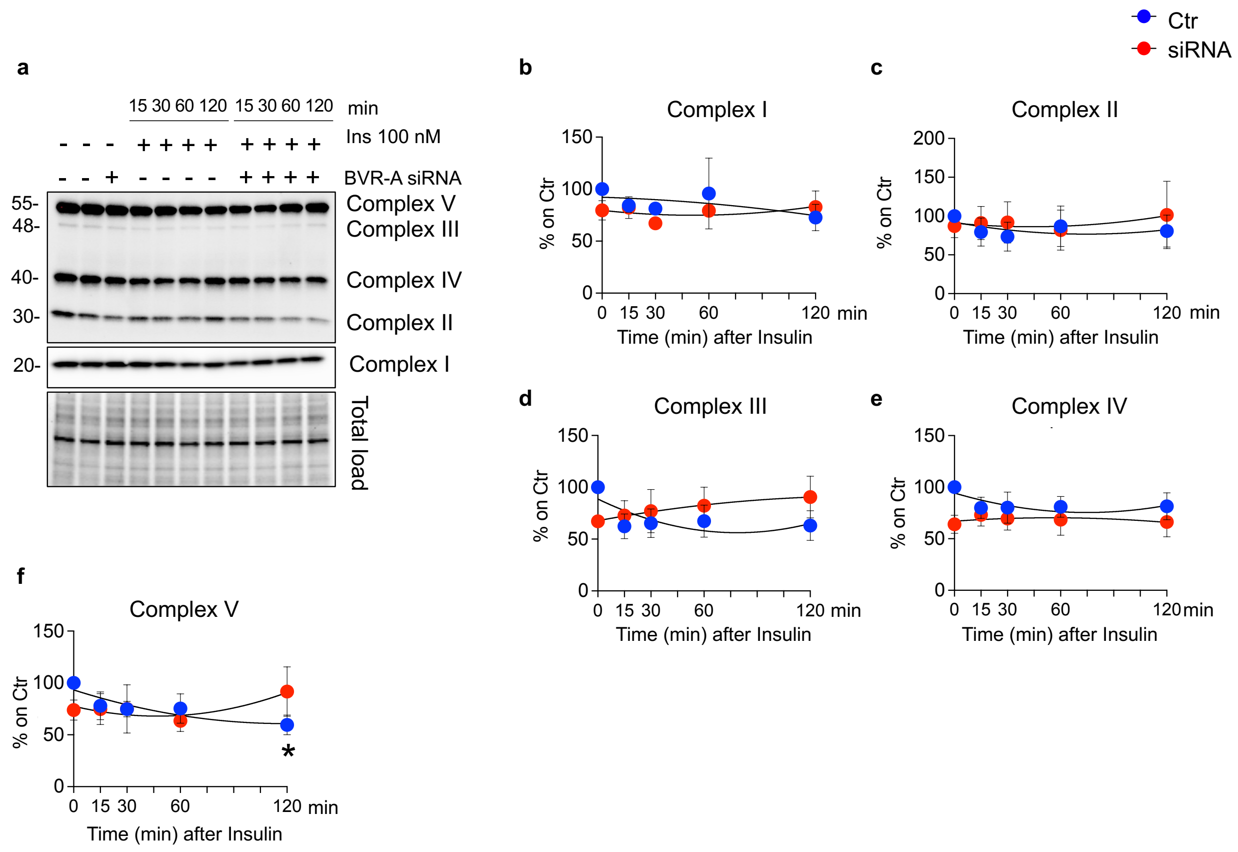

Supplementary Figure 4. Changes of mitochondrial complexes levels in insulin-stimulated SHSY-5Y cells

Changes of OXPHOS protein levels evaluated in Ctr and siRNA-treated cells stimulated with 100nM insulin at different time points 15', 30', 60' and 120' minutes (n =2-3 independent cultures/group). **(a)** Representative Western Blot images of OXPHOS in SHSY-5Y and densitometric evaluation of (**b)** Complex I (subunit NDUFB8), **(c)** Complex II (subunit SDHB), **(d)** Complex III (subunit UQCRC2), **(e)** Complex IV (subunit MTCO1), and **(f)** Complex V (subunit ATP5A), (*p=0.01, Ctr vs 120' Ins). Values are given as percentage of Ctr set as 100%. Values are given as percentage of Ctr 0' set as 100%. Data are presented as means ± SEM (One-way ANOVA with Fisher's LSD test). For differences between Ctr treated cells: *p<0.05, **p<0.01, ***p<0.001 and ****p<0.0001 and °p<0.05, °°p<0.01 for differences among Ctr and siRNA treated cell.

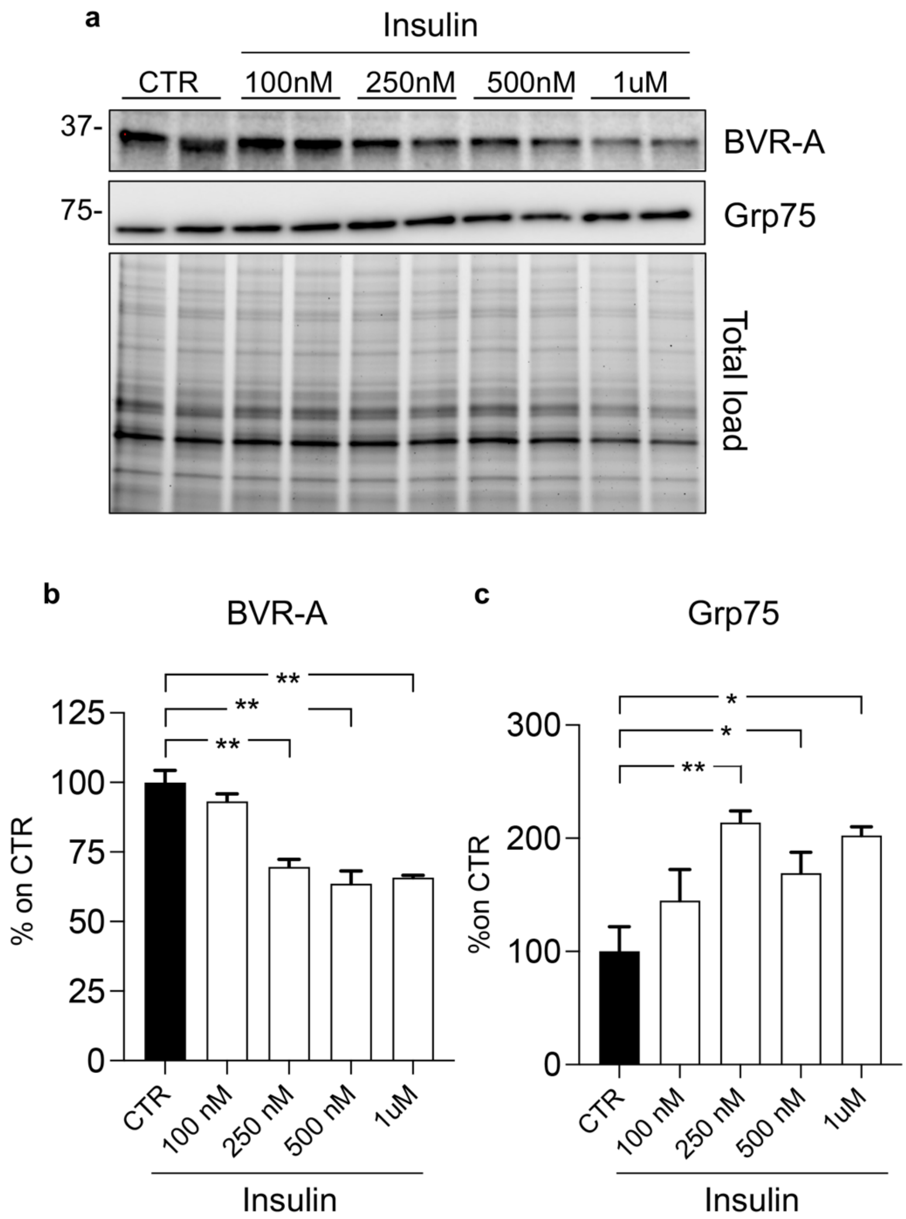

Supplementary Figure 5. Changes of BVR-A and Grp75 protein levels evaluated in SHSY-5Y cells in response to increasing doses of insulin

Changes of BVR-A and Grp75 protein levels in Ctr-treated cells stimulated with increasing concentrations of insulin (100nM, 250nM, 500nM, 1uM) for 30 minutes (n=2 independent cultures/group). (**a**) Representative Western blot images and **(b)** densitometric evaluation of BVR-A (p=0.0072, Ctr vs 250nM; p=0.00731 Ctr vs 500nM; p=0.0042, Ctr vs 1uM) and **(d)** Grp75 (p=0.0076, Ctr vs 250nM; p=0.04 Ctr vs 500nM; p=0.01, Ctr vs 1uM). Values are given as

percentage of Ctr set as 100%. Data are presented as means ± SEM (One-way ANOVA with Fisher's LSD test).

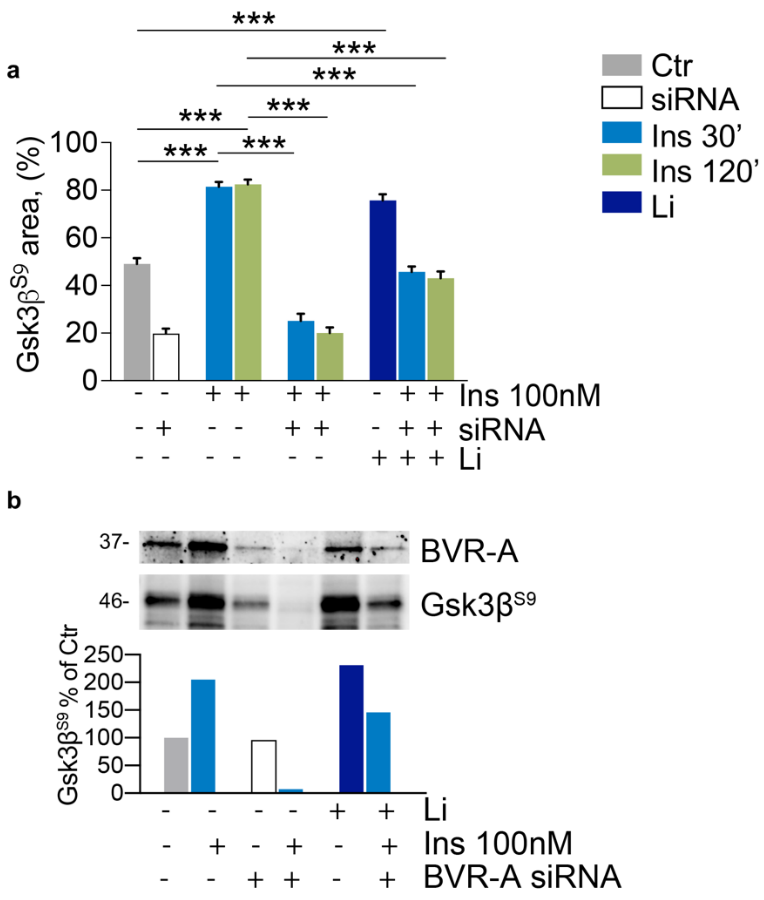

Supplementary Figure 6. Loss of BVR-A impairs insulin signaling activation and GSK3β translocation into the mitochondria in in SHSY-5Y cells in response to insulin

Bar chart showing the analysis of the confocal immunofluorescence images of GSK3β^{S9} signal over single cell area in Ctr and siRNA-treated cells stimulated with 100nM insulin for 30' and 120'. Where indicated cells were pre-treated with 10mM lithium chloride (LiCl) for 24 h before insulin administration. ($p<1E-15$, Ctr vs 30'Ins; $p<1E-15$, Ctr vs 120'Ins; $p<1E-15$, 30'Ins vs 30'siRNA+Ins; $p<1E-15$, 120'Ins vs 120'siRNA+Ins; $p<1E-15$, 30'Ins vs 30'siRNA+Ins+Li; $p<1E-15$, 120'Ins vs 120'siRNA+Ins+Li; $p=1.03E-11$ Ctr vs Ctr+Li). Values are given as mean ± SEM (n=12-18 cells from each condition from 3 independent experiments; One-way ANOVA with Sidak's multiple comparison test). **(b)** Western Blot of BVR-A and GSK3β^{S9} in mitochondrial

extraction from in Ctr and siRNA-treated cells stimulated with 100nM insulin for 30’ and 120’ **(c)** Densitometric evaluation of BVR-A and GSK3β^{S9} in mitochondrial isolated cells upon different treatments. Values are given as percentage of Ctr set as 100%.

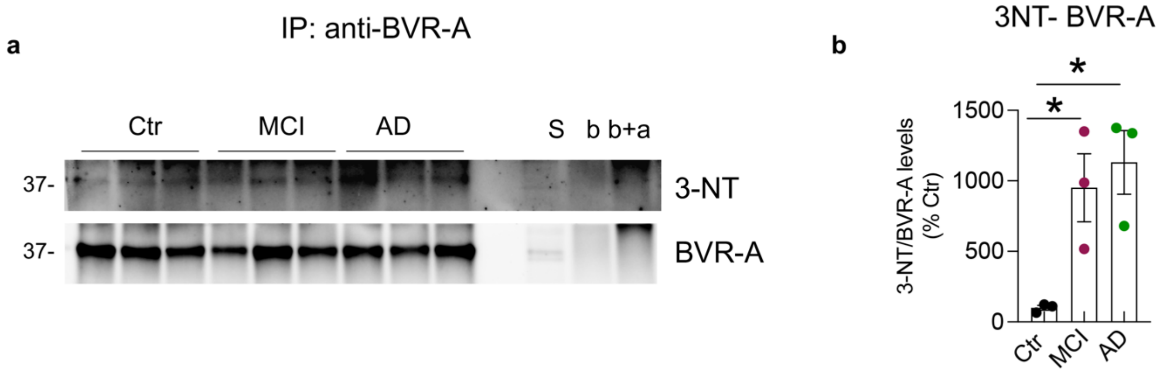

Supplementary Figure 7. BVR-A is target of nitrosative stress-induced damage in the IPL of patients with Mild Cognitive Impairment and Alzheimer's Disease

(a) Representative Western blot images and **(b)** densitometric evaluation of the 3-nitrotyrosine adducts on BVR-A (3-NT-BVR-A) isolated through the immunoprecipitation assay from the post-mortem IPL samples collected from Ctr, MCI and AD patients (n=3 per group, p=0.01, Ctr vs. MCI; p=0.008, Ctr vs. AD). Lanes description: lane 1-3: Ctr, lane 4-6: MCI samples, lane 7-9: AD samples, lane 10: empty, lane 11: Supernatant, lane 12: beads, lane 13: beads with primary anti-BVR-A antibody. All densitometric values are given as percentage of Ctr set as 100%. Data are presented as means ± SEM (One-way ANOVA with Fisher's LSD test).

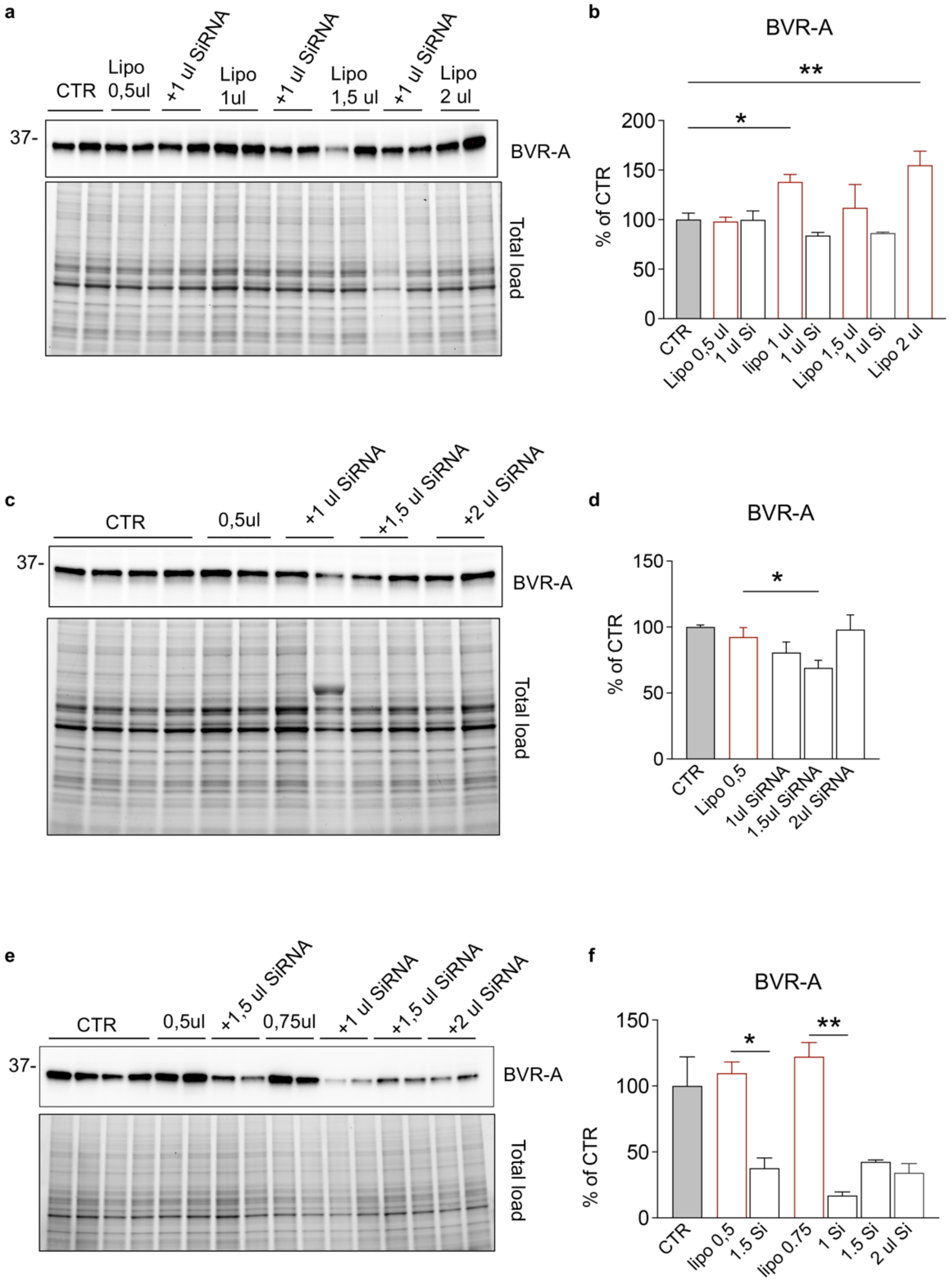

Supplementary Figure 8. Optimization of Lipofectamine Concentration for Efficient BVR-A silencing

(a,b) Evaluation of Lipofectamine concentration on transfection efficiency in SHSY-5Y cells. Different Lipofectamine concentrations (0.5 µl, 1 µl, 1.5 µl, and 2 µl) were tested alone or

alongside with 1 µl BVR-A siRNA to determine the efficiency grade of transfection. **(a)** Western blot and **(b)** densitometric evaluation of BVR-A. Notably, 0.5 µl of Lipofectamine alone did not significantly alter BVR-A protein levels when compared to Ctr cells and therefore was used for further analyses. **(c, d)** Assessment of BVR-A SiRNA concentration. Using 0.5 µl of Lipofectamine along with different concentrations of BVR-A siRNA (1 µl, 1.5 µl, and 2 µl). **(c)** Western blot and **(d)** densitometric evaluation of BVR-A. A significant reduction in BVR-A protein levels was observed with 0.5 µl of Lipofectamine + 1.5 µl BVR-A siRNA. **(e, f)** Validation of observed effects. The experiment was repeated using 0.5 µl of Lipofectamine and 1.5 µl of BVR-A siRNA, confirming a significant reduction in BVR-A protein levels. Additionally, an intermediate Lipofectamine volume (0.75 µl) was tested with varying SiRNA concentrations (1 µl, 1.5 µl, and 2 µl), revealing a significant reduction in BVR-A protein levels with 0.75 µl of Lipofectamine +1 µl siRNA. **(e)** Western blot and **(f)** densitometric evaluation of BVR-A. Data are presented as means ± SEM (One-way ANOVA with Fisher's LSD test).
